# The Non-receptor Tyrosine Kinase Tec Controls Assembly and Activity of the Noncanonical Caspase-8 Inflammasome

**DOI:** 10.1371/journal.ppat.1004525

**Published:** 2014-12-04

**Authors:** Florian Zwolanek, Michael Riedelberger, Valentina Stolz, Sabrina Jenull, Fabian Istel, Afitap Derya Köprülü, Wilfried Ellmeier, Karl Kuchler

**Affiliations:** 1 Department of Molecular Genetics, Medical University of Vienna, Max F. Perutz Laboratories, Vienna, Austria; 2 Division of Immunobiology, Institute of Immunology, Center for Pathophysiology, Infectiology and Immunology, Medical University of Vienna, Vienna, Austria; University of Pittsburgh, United States of America

## Abstract

Tec family kinases are intracellular non-receptor tyrosine kinases implicated in numerous functions, including T cell and B cell regulation. However, a role in microbial pathogenesis has not been described. Here, we identified Tec kinase as a novel key mediator of the inflammatory immune response in macrophages invaded by the human fungal pathogen *C. albicans*. Tec is required for both activation and assembly of the noncanonical caspase-8, but not of the caspase-1 inflammasome, during infections with fungal but not bacterial pathogens, triggering the antifungal response through IL-1β. Furthermore, we identify dectin-1 as the pathogen recognition receptor being required for Syk-dependent Tec activation. Hence, Tec is a novel innate-specific inflammatory kinase, whose genetic ablation or inhibition by small molecule drugs strongly protects mice from fungal sepsis. These data demonstrate a therapeutic potential for Tec kinase inhibition to combat invasive microbial infections by attenuating the host inflammatory response.

## Author Summary

Inflammasomes represent multi-protein complexes and their activation during microbial infections is key in driving hyperinflammation through the maturation and release of IL-1β, as well as by directly inducing several pro-inflammatory cytokines during the host pathogen interaction. Thus, inflammasomes are involved in the induction of pathogen-induced sepsis in mice and men. However, the precise regulation and molecules involved in triggering the assembly and activation of inflammasomes remain enigmatic. In this study, we identify the Tec Kinase family member Tec as a novel central regulator of the noncanonical caspase-8 inflammasome, regulated via the C-type lectin receptor dectin-1. Tec is involved in the assembly and activation of the caspase-8 inflammasome in response to the major human fungal pathogen *Candida albicans*, but not involved in anti-bacterial immune responses. Furthermore, Tec specifically regulates the caspase-8 inflammasome leaving the caspase-1 inflammasome unaffected. Finally, we are able to define Tec as novel molecular fine tuner of anti-fungal immune responses in a mouse model of acute sepsis. Strikingly, chemical inhibition of Tec with a small molecule drug also strongly protected mice from fungal sepsis. Hence, these data demonstrate a therapeutic potential for Tec kinase inhibition to combat invasive microbial infections by attenuating the host inflammatory response.

## Introduction

The host defense machinery of the innate immune system exploits highly dynamic mechanisms that respond and eliminate microbial infections. Cells of the innate immune system express germline-encoded pattern recognition receptors (PRRs). PRRs recognize pathogen-associated molecular patterns (PAMPs) to sense and detect microbial pathogens, subsequently orchestrating a controlled inflammatory immune response that is required for pathogen clearance [1], [2].

The hallmark pro-inflammatory cytokine interleukin-1β (IL-1β) is an important mediator of inflammatory responses during many microbial infections. It has been implicated in the pathophysiology of many infectious diseases as well as auto-immune disorders [3], [4]. Hence, expression as well as processing and release of active IL-1β are tightly controlled [5]. Inactive pro-IL-1β is synthesized in response to various stimuli, including bacterial, fungal and viral pathogens, resulting in an accumulation of intracellular pro-IL-1β. A second stimulus then triggers maturation of inactive pro-IL-1β into active IL-1β, which is released into the extracellular space via a non-classical secretory pathway, resulting in local or systemic inflammation and recruitment and activation of other migratory immune cells from distant body sites, including hematopoietic reservoirs such as bone marrow [6], [7]. Processing of inactive pro-IL-1β in response to infections or other inflammatory stimuli is mainly mediated by caspase-1, caspase-8 or caspase-11 through so-called inflammasomes [8]–[10]. Canonical inflammasomes contain the adaptor protein ASC and caspase-1 and/or caspase-11 and assemble upon stimulation of a cytosolic pattern recognition receptor such as NOD-like receptors (NLRs). By contrast, noncanonical inflammasomes are protein complexes lacking an activating cytosolic sensor molecule [8]. Notably, the activation of a noncanonical caspase-8 inflammasome upon fungal und mycobacterial stimuli has been reported. Strikingly, triggering of the C-type lectin receptor (CLR) dectin-1 leads to Syk-dependent formation of a CARD9–Bcl-10–MALT1 scaffold, which recruites and activates a MALT1–caspase-8–ASC-containing inflammasome that mediates IL-1β processing in response to *Candida albicans* [11]. However, signaling pathways and upstream molecules involved in the activation of the noncanonical caspase-8 inflammasome remain unknown.

The Tec kinase family represents the second-largest group of intracellular non-receptor tyrosine kinases. Five distinct family members are found exclusively in vertebrate hematopoietic cell lineages [12]. The Tec kinase family members Tec (tyrosine kinase expressed in hepatocellular carcinoma) and Btk (Bruton's tyrosine kinase) are expressed in cells of the myeloid as well as the lymphoid lineages [13]. Noteworthy, Btk has been implicated in B-cell and monocyte signaling [14], as well as in microbial phagocytosis [15]. However, Tec-dependent molecular functions and mechanisms, as well as a possible role during microbial infections remain elusive.

Here, we demonstrate a novel and hither unrecognized role for the Tec kinase in the innate immune response to pathogenic challenge. We decipher the pathway and mechanism through which Tec drives both assembly and activation of the noncanonical caspase-8 inflammasome in macrophages. Strikingly, we provide evidence that signaling via Tec is only involved in immune responses triggered by fungal but not by bacterial pathogens. The data identify Tec as a novel microbial signaling mediator in the dectin-1 pathway, acting downstream of Syk kinase and upstream of PLCγ2 in innate immune cells. Tec-deficiency causes a dramatic impairment of the inflammatory response upon challenge with the major human fungal pathogen *Candida albicans*. Moreover, the genetic removal of Tec leads to massively decreased caspase-8-mediated IL-1β processing *in vitro* and *in vivo*. Strikingly, using two different murine infection models, Tec-deficient mice are highly resistant to fungal sepsis. Hence, Tec kinase could be a suitable target for efficient antifungal therapy, aiming to reduce or diminish pathogen-induced hyperinflammation and/or sepsis. Our work also provides compelling support for novel antifungal therapeutic approaches, as well as for drug discovery, such that inhibiting host hyperinflammatory genes by small molecules may prove a suitable strategy to combat fatal invasive fungal diseases.

## Results

### Tec is required to mount inflammatory immune responses

**Figure 1:**
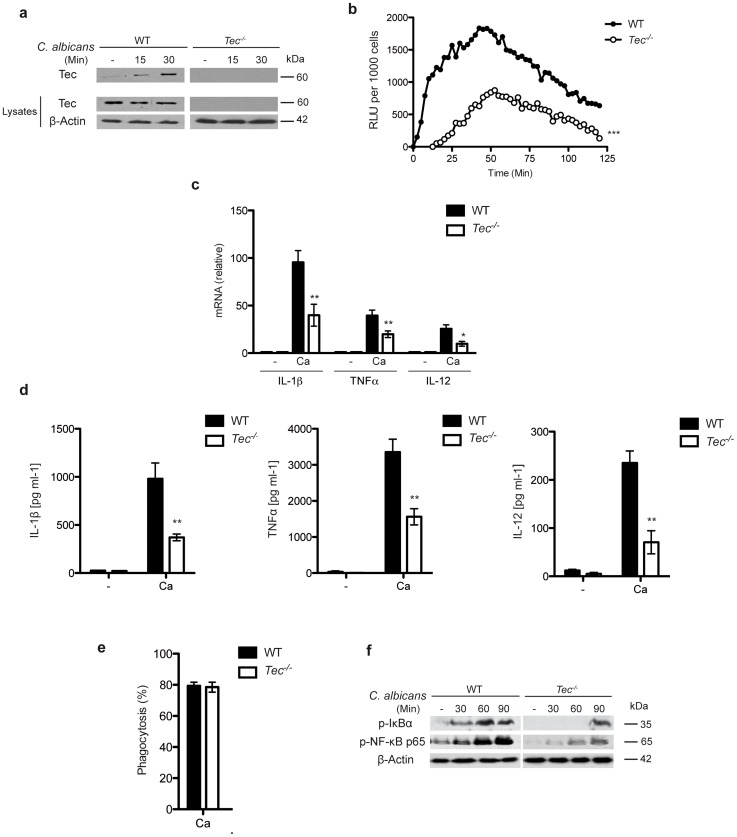
Lack of Tec impairs the inflammatory response to fungal pathogens. (**a**) Immunoblot analysis of Tec and (**b**) qPCR analysis of Tec expression after stimulating BMMs with *C. albicans* for 120 min; results are normalized to GAPDH (glyceraldehyde phosphate dehydrogenase). (**a**) Immunoblot of activated Tec in cell lysates after stimulation with *C. albicans*; lysates were enriched for phospho-proteins. (**b**) Detection of reactive oxygen species from BMMs after *C. albicans* challenge for 120 Min using luminol (ROS from unstimulated cells was subtracted). (**c**) qPCR analysis of cytokine response after 120 Min without (-) or with stimulation with *C. albicans* (Ca); results are normalized to GAPDH. (**d**) ELISA for cytokines in supernatants of BMMs with or without (-) *C. albicans* (Ca) stimulation. (**e**) Rate of phagocytosis after 45 Min of incubation with *C. albicans* (Ca). (**f**) Immunoblotting of p-IκBα and p-NF-κB p65 activation over the time course of *C. albicans* infection in BMMs. Data are representative of at least two (**a**–**c**, **g**) or three (**d**–**f**) independent experiments. Mean and SD are shown.

Tec kinase is expressed in the myeloid lineage, including dendritic cells, neutrophils and macrophages [13]. Notably, lack of Tec did not affect *in vitro* differentiation of murine bone marrow-derived macrophages (BMMs) (**Fig. S1a**), as previously shown [16]. In addition, challenge with the pathogenic fungus *Candida albicans* did not affect Tec expression levels (**Fig. S1b**), but activated the kinase as evident from its tyrosine phosphorylation (**Fig. 1a**) in BMMs. We next investigated whether Tec is involved in the inflammatory response and whether loss of Tec would cause any significant changes. For instance, phagocytosis and subsequent production of reactive oxygen species (ROS) and inflammatory cytokines constitutes a hallmark defense to microbial challenge [17]. Strikingly, ROS production was also strongly diminished when challenged by Candida spp (**Fig. 1b**). Likewise, expression and release of the hallmark inflammatory cytokines IL-1β, TNFα and IL-12 was strongly reduced (**Fig. 1c,d**). Additionally, Tec-deficient BMMs showed severe MOI-independent impairments in IL-12 and TNFα release (**Fig. S1c**), although leaving fungal phagocytosis unaffected (**Fig. 1e**). Of note, stimulation of BMMs with zymosan or curdlan, two classical fungal PAMP-mimics also exhibited a reduced inflammatory response, as indicated by decreased levels of TNFα. Furthermore, stimulation with the TLR2 agonist Pam_3_CSK_4_ revealed reduced production of TNFα in Tec-deficient cells (**Fig. S1d**). Interestingly, however, stimulation with bacterial PAMP lipopolysaccharide (LPS) was not altered (**Fig. S1d**). Surprisingly, the impaired inflammatory response was not due to defective signaling through the mitogen-activated kinases (MAP-Kinase) ERK or p38 (**Fig. S1e**). However, activation of NF- κB was clearly decreased (**Fig. 1f**). Thus, the lack of the non-receptor tyrosine kinase Tec severely impairs the inflammatory signaling response to fungal pathogens.

### Tec activates caspase-8-dependent IL-1β processing

**Figure 2:**
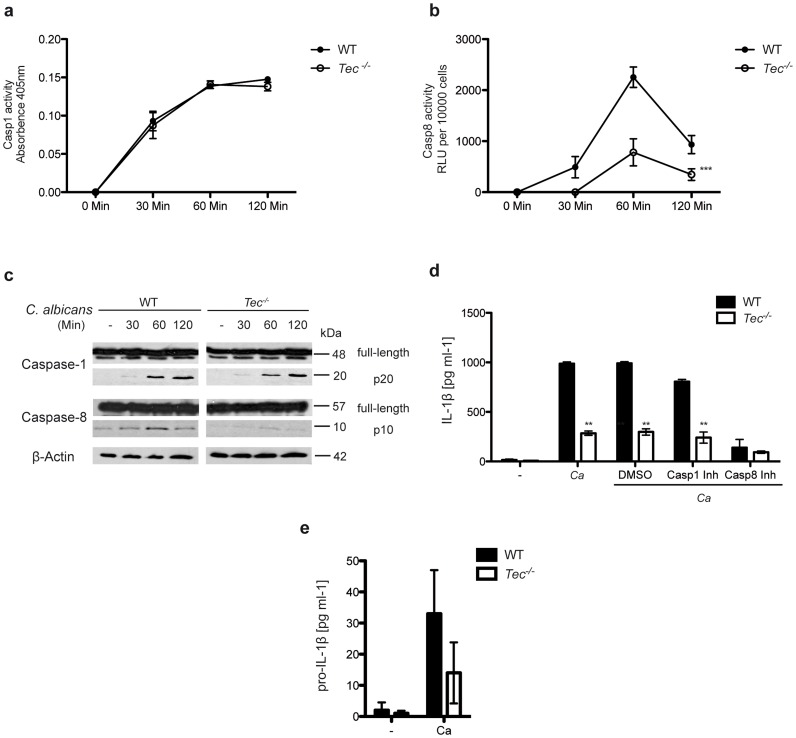
Caspase-8 activity in response to *C. albicans* requires Tec in BMMs. (**a**) Caspase-1 activity over the course of infection with *C. albicans*; absorbence of unstimulated cells and *C. albicans* only was subtracted. (**b**) Caspase-8 activity over the course of infection with *C. albicans*; chemiluminenscence of unstimulated cells and *C. albicans* only was subtracted. (**c**) Immunoblot analysis of full-length or active (p20) caspase-1 and full-length and active (p10) caspase-8 during the course of BMM infection with *C. albicans*. (**d**) ELISA of IL-1β in supernatants of BMMs after stimulation with *C. albicans* only (Ca) or with dimethylsulfoxide (DMSO), Casp1 inhibitor (Casp1 Inh; 5 mM) or Casp8 inhibitor (Casp8 Inh; 5 mM) and Ca or left unstimulated (-). (**e**) ELISA of pro-IL-1β in supernatants of BMMs after stimulation with *C. albicans* (Ca) or left unstimulated (-). Data are representative of at least two (**c**), three (**d,e**) or five (**a**,**b**) independent experiments. Mean and SD are shown.

Processing and release of mature IL-1β requires the activity of distinct caspase proteases [8]–[10]. Therefore, we wanted to quantify the activities of caspase-1 and caspase-8 in response to fungal challenge. Tec-deficient BMMs revealed normal activity of caspase-1, but dramatically reduced levels of active caspase-8 (**Fig. 2a,b**). These results were also confirmed by immunoblotting, detecting the active subunits of caspase-1 and caspase-8 (**Fig. 2c**). Blocking caspases by specific chemical inhibitors also revealed reduced activity of caspase-8 but not of any other caspases tested (**Fig. S2a,b**), indicating a lack of crosstalk between the two pathways. In addition, while the release of mature IL-1β was caspase-1 independent, it showed a strong dependency on caspase 8 activity (**Fig. 2d**). Noteworthy, the analysis of the intracellular pro-IL-1β precursor levels after fungal challenge revealed that Tec-deficient cells showed a tendency of reduced pro-IL-1β, albeit not reaching significance. This data could reflect reduced transcriptional levels as observed for IL-1β (**Fig. 2e**), but further imply complex post-translational as well as post-transcriptional processes modulating the release kinetics of extracellular IL-1β.

To exclude global defects in caspase activity or a possible impaired apoptosis in *Tec^−/−^* cells, we also determined the activities and cleavage of caspase-3, caspase-7, caspase-9, as well as of poly ADP ribose polymerase (PARP) in response to *Candida albicans*. However, no differences were observed between wild-type and *Tec^−/−^* knock-out macrophages (**Fig. S2c,d**). Hence, these data suggest a specific and exclusive role for Tec in the caspase-8 dependent IL-1β response upon pathogenic challenge.

### Assembly of the caspase-8 inflammasome requires both Tec & Syk signaling

**Figure 3:**
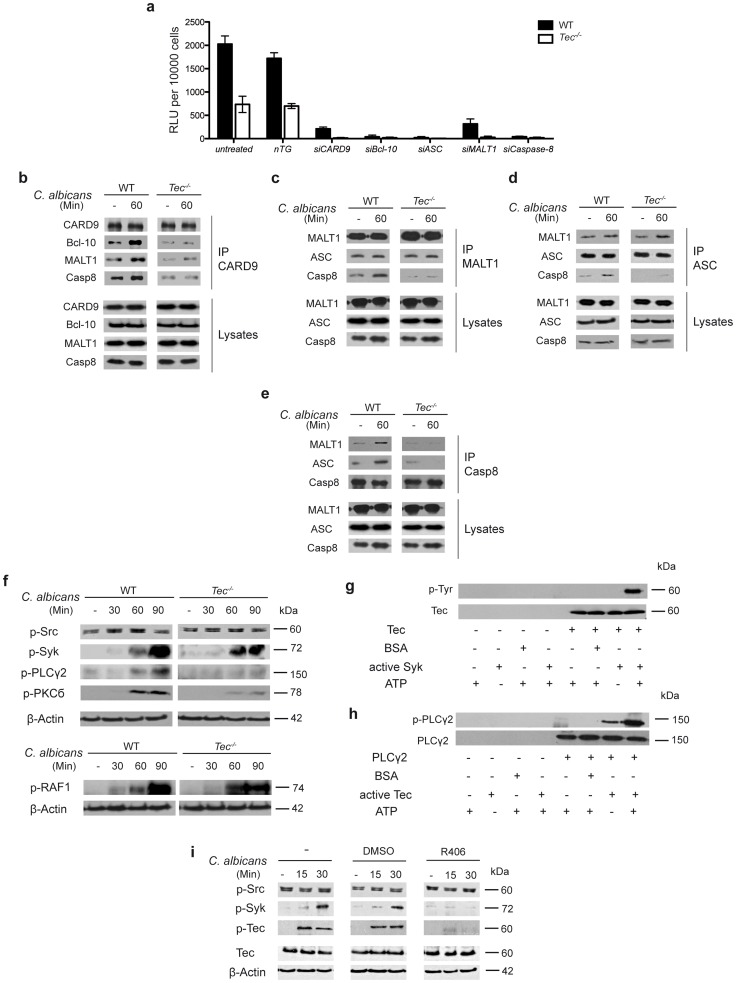
Tec is required for the assembly of the caspase-8 inflammasome. (**a**) Caspase-8 activity after 60 Min of stimulation with *C. albicans* of cells left untreated, knockdown of a non-target (nTG; 25 nM) or respective siRNA knock down (25 nM) after 72 hrs of incubation; chemiluminenscence of unstimulated cells and *C. albicans* only was subtracted. (**b**–**e**) Immunoblot analysis of CARD9, Bcl-10, MALT1, ASC and caspase-8 (Casp8) after immunoprecipitation (IP) with antibodies against CARD9 (**b**), MALT1 (**c**), ASC (**d**) and caspase-8 (**e**) from whole-cell lysates of BMMs left unstimulated (-) or stimulated with *C. albicans* for 60 Min. Data are representative of two independent experiments for each IP. (**f**) Immunoblot analysis of p-Src, p-Syk, p-PLCγ2, p-PKCδ and p-RAF1 during the course of BMM infection with *C. albicans*. (**g**) *In vitro* kinase assay; Tec was immunoprecipitated from unstimulated BMMs and incubated with recombinant active Syk, BSA (70 ng each) and adenosine triphosphate (ATP, 200 nM) for 30 Min at 30°C; active phosphorylated Tec was detected with α-p-Tyr antibodies. (**h**) *In vitro* kinase assay; PLCγ2 was immunoprecipitated from unstimulated BMMs and incubated with active Tec, BSA (70 ng each) and adenosine triphosphate (ATP, 200 nM) for 30 Min at 30°C; active PLCγ2 was detected with α-p-PLCγ2 antibody. (**i**) Immunoblot of activated Tec and p-Src/p-Syk in cell lysates after stimulation with *C. albicans* and parallel Syk inhibition with R406 (3 µM); lysates were enriched for phospho-proteins. Data are representative of at least two (**b**–**i**) or three (**a**) independent experiments. Mean and SD are shown (**a**).

An elegant recent paper reported about a noncanonical caspase-8 inflammasome that processes pro-IL-1β into the mature cytokine in response to curdlan in human dendritic cells [11]. We therefore examined whether all of the necessary components for interleukin processing were also operating in murine BMMs. Indeed, CARD9, Bcl-10, MALT1, ASC and caspase-8 were expressed in macrophages but their levels were unaltered upon fungal challenge (**Fig. S3a**). To verify whether the noncanoncial caspase-8 inflammasome was functional, we decided to knock-down each component of the machinery and test the activation of caspase-8 after stimulation with *Candida albicans* (**Fig. S3b,c**). In essence, knock-down of each single component led to a significant decrease in caspase-8 activity (**Fig. 3a**). Thus, the noncanonical caspase-8 inflammasome is fully functional in murine BMMs.

Next, we tested whether Tec-deficient macrophages exhibited any impairment in the assembly of the caspase-8 inflammasome. We immunoprecipitated all components of the inflammasome complex following fungal stimulation. We stimulated BMMs for 60 minutes with *Candida albicans*, since this was the time point showing highest caspase-8 activity (**Fig. 2b**). Strikingly, *Tec^−/−^* cells showed a severely impaired assembly of the inflammasome scaffold components such as CARD9, Bcl-10 and MALT1 (**Fig. 3b**). Moreover, we detected defects in the assembly of the caspase-8 inflammasome, consisting of MALT1, ASC and caspase-8, for each of the components used for immunoprecipitation in Tec-deficient cells (**Fig. 3c–e**). Interestingly, we noticed that MALT1 and ASC seemed to pre-associate without any pathogen stimulus. Neither fungal stimulation nor Tec-deficiency affected MALT1 and ASC interaction (**Fig. 3c,d**), implying that these two components may constantly associate in murine BMMs. Thus, these data demonstrate that Tec kinase is required for both assembly and activation of the noncanonical caspase-8 inflammasome in response to *Candida albicans*.

Signaling through and assembly of the noncanonical caspase-8 inflammasome requires Syk as well as CARD9 [8] and several signaling pathways have been reported to activate CARD9 in response to fungal challenge [18]–[21]. Hence, we tested whether the non-receptor tyrosine kinase Tec was involved in relevant signaling cascade. Tec-deficient BMMs revealed normal activation patterns for both Src and Syk kinases, as well as of c-RAF in response to *Candida albicans* (**Fig. 3f**). By contrast, striking differences were seen in the activation of PLCγ2 and PKCδ (**Fig. 3d**), demonstrating that Tec must be acting downstream of the Syk-kinase but upstream of PLCγ2, as previously shown for osteoclasts and mast cells [22], [23].

To further confirm this biochemically, we performed *in vitro* kinase assays. Indeed, recombinant, active Syk-kinase activated Tec *in vitro* (**Fig. 3g**). Likewise, recombinant Tec activated downstream PLCγ2 (**Fig. 3h**). Notably, we were able to detect PLCγ2 activation even without ATP, suggesting that active Tec is necessary and sufficient to activate PLCγ2; addition of ATP only led to an increase of this reaction. Finally, we addressed the question whether Syk inhibition decreases Tec activation. Remarkably, treatment of cells with the specific Syk inhibitor R406 significantly diminished activation of Tec in response to *Candida albicans* (**Fig. 3i**). Taken together, these data show that Tec kinase operates in the signaling pathway that drives assembly of the caspase-8 inflammasome. Fungal challenge mediates Tec activation by Syk and thereafter leads to the phosphorylation of PLCγ2 to drive inflammasome activation.

### Tec couples dectin-1 signaling to caspase-8 activation

**Figure 4:**
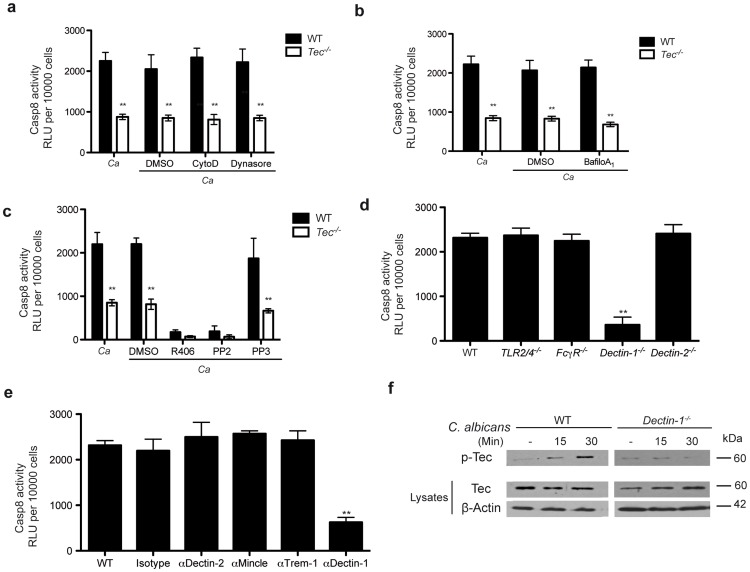
Dectin-1 is required for Tec-dependent caspase-8 activation. (**a**) Caspase-8 activity after 60 Min stimulation of BMMs with *C. albicans* (Ca), dimethylsulfoxide (DMSO), CytochalasinD (CytoD; 2 µM) or Dynasore (80 µM); chemiluminenscence of unstimulated cells, cells with respective inhibitor, cells with DMSO or *C. albicans* only was subtracted. (**b**) Caspase-8 activity after 60 Min of stimulation with *C. albicans* only (Ca) or with dimethylsulfoxide (DMSO) or BafilomycinA_1_ (BafiloA_1_; 30 nM) and Ca; chemiluminenscence of unstimulated cells, cells with respective inhibitor, cells with DMSO or *C. albicans* only was subtracted. (**c**) Caspase-8 activity after 60 Min of stimulation with *C. albicans* only (Ca) or with dimethylsulfoxide (DMSO), Syk Inhibitor R406 (3 µM), Src Inhibitor PP2 (5 µM) or the non-functional analogon PP3 (5 µM) and Ca; chemiluminenscence of unstimulated cells, cells with respective inhibitor, cells with DMSO or *C. albicans* only was subtracted. (**d**) Caspase-8 activity after 60 Min stimulation with *C. albicans* only (Ca) in BMMs of indicated genotype; chemiluminenscence of unstimulated cells or *C. albicans* only was subtracted. (**e**) Caspase-8 activity after 60 Min of stimulation with *C. albicans* only (Ca) in WT BMMs blocked with indicated antibodies (all 10 µg/ml) and respective isotype control (10 µg/ml); chemiluminenscence of unstimulated cells, cells treated with antibody only or *C. albicans* only was subtracted. (**f**) Immunoblot of Tec activation of cell lysates after stimulation with *C. albicans*; lysates were enriched for phospho-protein fraction using respective kit. Data are representative of at least two (**f**), three (**d**,**e**) or four (**a**–**c**) independent experiments. Mean and SD are shown.

We also assessed whether caspase-8 activity in response to fungal challenge in murine macrophages requires phagocytosis. However, and interestingly, the use of the chemical phagocytosis inhibitors Cytochalasin D and Dynasore demonstrated that caspase-8 activation and release of mature IL-1β was fully phagocytosis-independent (**Fig. 4a**; **Fig. S4a**). Furthermore, the use of Bafilomycin A_1_ showed that phagosomal acidification was not necessary for inflammasome activation (**Fig. 4b**; **Fig. S4b**). However, we found that signalling via Syk- and Src-kinases was essential for *Candida*-induced caspase-8 activity, as well as IL-1β maturation, since inhibition of Syk with the specific inhibitor R406 and inhibition of Src with PP2 completely abolished both caspase activity and cytokine release (**Fig. 4c**; **Fig. S4c**). Noteworthy, a block of phagocytosis or phagosomal acidification left levels of pro-IL-1β unaffected. However, inhibition of Src- and Syk-kinases decreased the intracellular pro-IL-1β precursor levels when compared to untreated controls (**Fig. S4d**).

Several pathogen recognition receptors are involved in antifungal immune responses, including members of the toll-like family (TLRs) and the C-type lectin family (CLRs) [24]–[26]. We therefore were curious whether any member of these families were also involved in triggering assembly of the caspase-8 inflammasome to drive processing of IL-1β. Since caspase-8 activation was phagocytosis-independent, we excluded any contribution of phagosomal TLRs to the activation of caspase-8. Furthermore, *Tlr2^−/−^/Tlr4^−/−^* BMMs confirmed no contribution of these TLR receptors, both of which have been otherwise implicated in fungal host response [24]–[26].

Hence, we therefore tested the role of C-type lectins. Neither FcγR-deficient nor *Dectin-2^−/−^* BMMs displayed impaired caspase activity or cytokine release in response to *Candida albicans*. However, *Dectin-1^−/−^* cells exhibited a dramatic decrease in inflammasome activation and IL-1β production (**Fig. 4d**; **Fig. S4e**). Moreover, inhibition of additional CLRs such as Mincle, Trem-1, Dectin-2 with respective blocking antibodies reconfirmed a detrimental role for dectin-1 (**Fig. 4e**; **Fig. S4f**).

We next performed immunoprecipitation experiments to assess whether the loss of dectin-1 in BMMs would impact the assembly of a *Candida*-induced caspase-8 inflammasome. Strikingly, *Dectin-1^−/−^* cells showed decreased assembly of the inflammasome scaffold consisting of CARD9, Bcl-10 and MALT1 (**Fig. S5a**). Furthermore, immunoprecipitation of inflammasome components such as ASC, MALT1 and caspase-8 verified defects in the assembly of the inflammasome in *Dectin-1*-deficient cells (**Fig. S5b**). These data phenocopy the results observed in *Tec^−/−^* BMMs. Of note, the pre-assembly of ASC and MALT1 was also observed in these knock-out cells, further arguing that ASC an MALT1 in murine BMMs might form a pre-inflammasome complex.

Finally, we wanted to assess whether dectin-1 and the non-receptor tyrosine kinase Tec are acting in the same pathway. We therefore decided to check activation of Tec in *Dectin-1*-deficient BMMs. Indeed, we detected a strongly diminished Tec activation in *Dectin-1^−/−^* BMMs upon *Candida albicans* challenge (**Fig. 4f**). Furthermore, we wanted to determine the contribution of dectin-1 signaling during the caspase-8-dependent activation of NF-κB. Hence, we stimulated WT and *Tec^−/^* ^−^ cells with the dectin-1 agonist curdlan in the presence of a chemical caspase-8 inhibitor. Interestingly, blocking of caspase-8 activity drastically decreased NF-κB activation in WT macrophages but not in Tec-deficient cells (**Fig. S5c**). Taken together, these data yield compelling evidence that dectin-1 signaling activates Tec following pathogen detection. Moreover, dectin-1 is the main PRR mediating the activation of the caspase-8 inflammasome in murine BMMs during fungal challenge.

### Fungal β-glucans activate caspase-8 through Tec signaling

**Figure 5:**
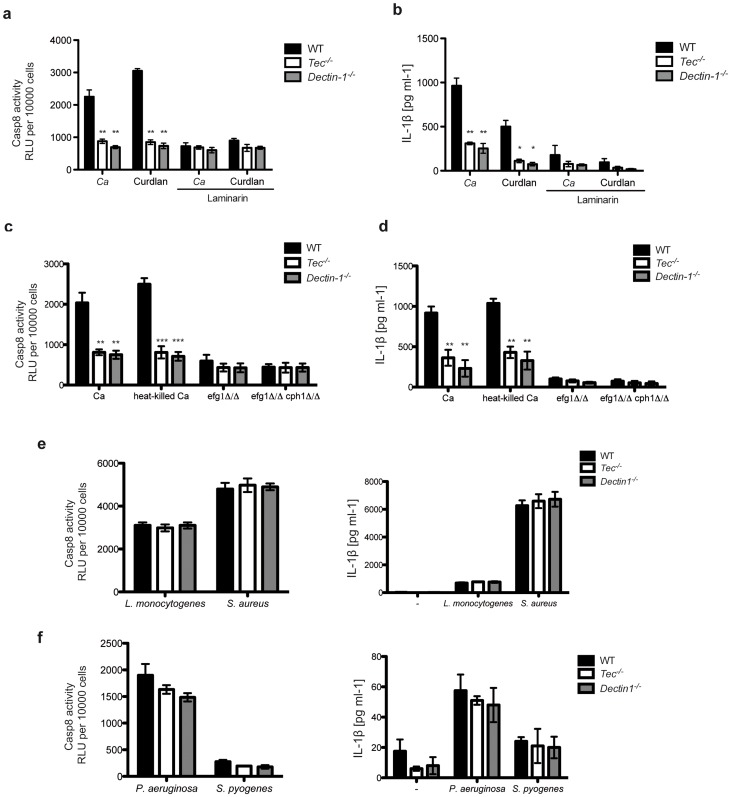
Fungal β-glucans activate caspase-8 in murine BMMs. (**a**) Caspase-8 activity after 60 Min stimulation of BMMs with *C. albicans* (Ca) or curdlan (200 µg/ml); dectin-1 was blocked with laminarin (500 µg/ml); chemiluminenscence of unstimulated cells, cells with laminarin only or *C. albicans* only was subtracted. (**b**) ELISA of IL-1β in supernatants of BMMs after stimulation with *C. albicans* only (Ca) or curdlan (200 µg/ml); dectin-1 was blocked with laminarin (500 µg/ml). (**c**) Caspase-8 activity after 60 Min of stimulation with *C. albicans* only (Ca), heat-killed Ca (10 Min on 70°C)); chemiluminenscence of unstimulated cells or *C. albicans* resp. *Candida glabrata* only was subtracted. (**d**) ELISA of IL-1β in supernatants of BMMs after stimulation with *C. albicans* only (Ca), heat-killed Ca (10 Min on 70°C), *efg1*Δ/Δ or *efg1*Δ/Δ *cph1*Δ/Δ Ca-mutants or left unstimulated (-). (**e,f**) Caspase-8 activity after 60 Min of stimulation with *L. monocytogenes*. *S. aureus*, *P. aeruginosa* or *S. pyogenes*; chemiluminenscence of unstimulated cells or bacteria only was subtracted. ELISA of IL-1β in supernatants of BMMs after stimulation with *L. monogenes*. *S. aureus*, *P. aeruginosa* or *S. pyogenes* or left unstimulated (-). Data are representative of at least three independent experiments. Mean and SD are shown.

The C-type lectin receptor dectin-1 recognizes β-glucans exposed on the cell surface of fungal pathogens [27]. Noteworthy, in living cells, β-glucans are normally masked by the outer layer mannans and only exposed on the hyphal tip of *Candida albicans* during its morphological transition from yeast to hyphae [28]. Hence, we wanted to address the question whether Tec transmits signals triggered by hyphae, and whether β-glucans represent the major PAMP for the activation of the *Candida*-induced caspase-8 inflammasome. We therefore decided to stimulate BMMs with curdlan as a PAMP mimic. Interestingly, curdlan exposure to *Tec-* and *Dectin-1*-deficient BMMs revealed striking differences in caspase-8 activities, as well as in the release of IL-1β when compared to WT control cells. Moreover, inhibition by laminarin of dectin-1 signaling in *Candida-* and curdlan-stimulated wild type BMMs almost abolished inflammasome activity, and reduced cytokine-release to knockout-levels. Both *Tec^−/−^* and *Dectin^−/−^* knock-out cells did not show any further decrease in caspase-8 activity upon laminarin inhibition (**Fig. 5a,b** and **Fig. S6b**).

Next, we decided to stimulate cells with heat-killed *Candida albicans*, since this treatment strongly increases β-glucan exposure on the cell surface [29]. In line with results observed with the synthetic ligand curdlan, *Tec^−/−^* and *Dectin-1^−/−^* cells showed drastically decreased caspase-8 activity and IL-1β release when compared to stimulated WT BMMs (**Fig. 5c,d** and **Fig. S6b**).

Finally, to address the question whether hyphae with hyper-exposed β-glucans fuel caspase-8 activity, we generated a yeast-locked *Candida albicans* strain, which is unable to form hyphae and is avirulent [30]. For this purpose, we genetically deleted Efg1 and Cph1, two fungal transcription factors essential for filamentation [31], [32]. As expected, *efg1*Δ/Δ as well as *efg1*Δ/Δ *cph1*Δ/Δ double mutants were unable to form hyphae under otherwise hyphae-inducing conditions (**Fig. S6a**). Strikingly, macrophages challenged with *efg1*Δ/Δ or *efg1*Δ/Δ *cph1*Δ/Δ *Candida albicans* mutants revealed only a minor activation of the caspase-8 inflammasome (**Fig. 5c**), which is consistent with an almost undetectable IL-1β release (**Fig. 5d**) and even significantly reduced levels of pro-IL-1β (**Fig. S6b**).

To further elucidate the role of Tec in the caspase-8-dependent processing of IL-1β, we stimulated BMMs with various fungal species. We therefore analysed the caspase-8 activity and IL-1β release in BMMs following challenge with several clinical fungal isolates, including *Candida albicans*, *Candida glabrata*, *Candida krusei*, *Candida lusitaniae* as well as *Cryptococcus neoformans* (**Fig. S7**). Interestingly, nearly all strains tested showed strong Tec- as well as dectin-1-dependent caspase-8 activity paired with IL-1β release. Noteworthy, different species and even different strains of the same species triggered quite different responses, which is most likely due to different levels of surface β-glucans or its content in respective strains. Strikingly, however, neither Tec-deficient nor dectin-1-deficient BMMs infected with different *Gram*-positive (**Fig. 5e**) or *Gram*-negative (**Fig. 5f**) bacteria displayed any changes in caspase-8 activity or IL-1β release compared to wild-type cells.

Taken together, these data demonstrate that surface-exposed β-glucans activate the caspase-8 inflammasome, leading to the processing and release of pro-inflammatory IL-1β in a Tec- and dectin-1-dependent manner.

### Tec is involved during fungal pathogenesis *in vivo*

**Figure 6:**
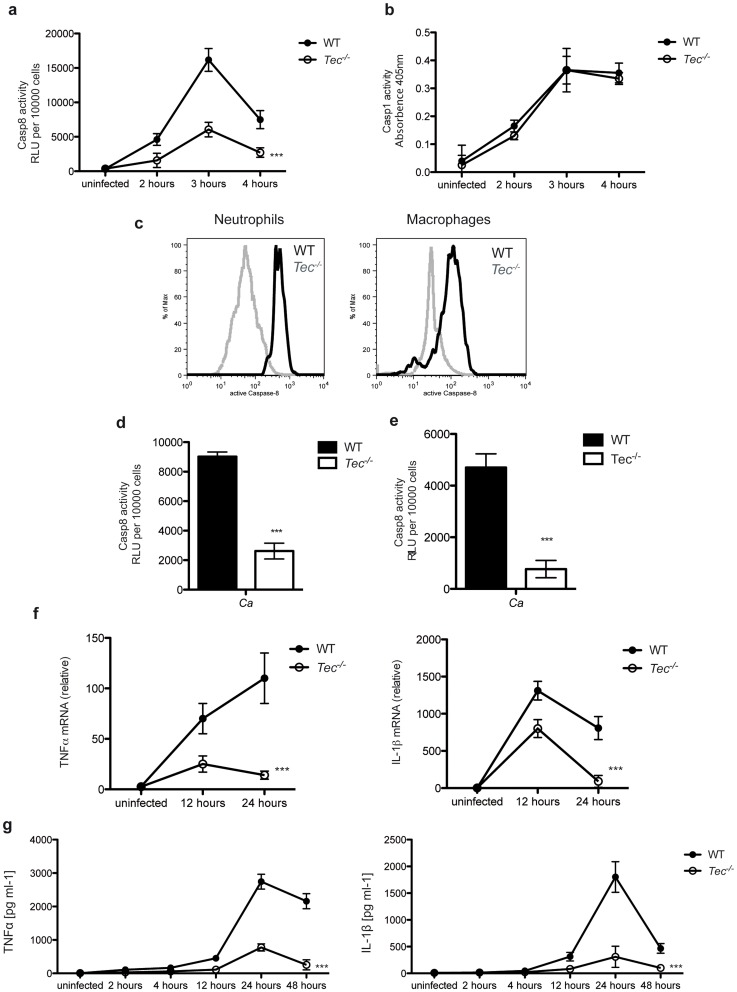
Tec regulates fungal virulence *in vivo*. (**a**) Caspase-8 activity of total murine peritoneal lavage cells obtained after intraperitoneal infection (*i.p.*) with 5.10^6^ CfUs of *C. albicans* after indicated times of infection or control lavage cells from uninfected mice; n = 3 per genotype and time point (**b**) Caspase-1 activity of total murine peritoneal cells upon *i.p.* infection with 5.10^6^ CfUs of *C. albicans* after indicated times of infection or cells from uninfected mice; n = 3 per genotype and time point (**c**) Intracellular staining of active caspase-8 in neutrophils (CD11b^+^Ly6C^+^Ly6G^+^) and macrophages (CD11b^+^F4/80^+^) in peritoneal lavage cells obtained after i.p. infection with 5.10^6^ CfUs of *C. albicans* after 24 h. (**d**) Mice were infected *i.p.* with 3% brewer thioglykolate medium for 4 days; lavage cells were collected; total caspase-8 activity was measured after 60 Min of stimulation with *C. albicans* (Ca); chemiluminenscence of unstimulated cells or *C. albicans* only was subtracted; n = 4 per genotype and time point (**e**) Polymorph-nuclear neutrophils were isolated from bone marrow of respective mice using a Percoll-gradient; Caspase-8 activity was measured after 60 Min of stimulation with *C. albicans* (Ca); chemiluminenscence of unstimulated cells or *C. albicans* only was subtracted. (**f**) qPCR analysis of TNFα and IL-1β of total murine peritoneal cells upon intraperitoneal infection (*i.p.*) with 5.10^6^ CfUs of *C. albicans* after indicated time of infection or cells from uninfected mice; results are normalized to those of GAPDH. n = 3 per genotype and time point (**g**) ELISA of TNFα and IL-1β in lavage of mice upon intraperitoneal infection (*i.p.*) with 5.10^6^ CfUs of *C. albicans* after indicated time of infection or from uninfected mice. n = 3 per genotype and time point. Data are representative of at least two (**c**) three (**a,b, e–g**) or four (**d**) independent experiments. Mean and SD are shown.

Till today, a role for Tec in microbial virulence or pathogenesis *in vivo* has remained elusive. To test whether Tec was involved in immune responses *in vivo*, we decided to use mouse models suitable to assess local inflammation and sepsis. After injection of *Candida albicans* into the peritoneum of mice, numbers and cell types of recruited immune cells into the peritoneum of WT or knockout mice were comparable (**Fig. S8a**). However, Tec-deficient mice revealed significantly reduced levels of caspase-8 activity in peritoneal immune cells obtained by lavage, more than 90% of which were in fact neutrophils (**Fig. 6a** **; Fig. S8a**). In contrast, same cells did reveal an unaltered caspase-1 response (**Fig. 6b**). To elucidate which immune cell-type showed the highest caspase-8 activity and in which loss of Tec affected caspase-8 activity the most in the peritoneum, we stained active caspase-8 intracellularly. Strikingly, neutrophils showed the highest levels of active caspase-8 and Tec-deficiency led to drastic reduction within these cells (**Fig. 6c**). To further quantify the contribution of peritoneal macrophages to the observed phenotype, mice were injected intraperitoneally with thioglykolate. After 4 days, lavage cells were isolated. At this time point, almost all recruited cells were peritoneal macrophages. Notably, a recruitment defect due to Tec-deficiency was also not observed here (**Fig. S8b**). Strikingly, however, peritoneal macrophages had strongly reduced levels of caspase-8 activity after re-stimulation with *Candida albicans in vitro* (**Fig. 6d**), confirming results obtained with BMMs also *in vivo*. Furthermore, we isolated polymorph-nuclear neutrophils from bone marrow of mice. Interestingly, neutrophils also displayed defects in the activation of the caspase-8 inflammasome (**Fig. 6e**). Next, we wanted to assess whether the reduced caspase-8 would impact the inflammatory response in a mouse model of fungal virulence. Indeed, we detected strongly reduced levels of the inflammatory cytokines at the RNA level in peritoneal cells (**Fig. 6f**). These results were confirmed by highly reduced protein levels of TNFα and IL-1β present in lavages of infected WT and *Tec^−/−^* mice (**Fig. 6g**). Of note, pro-IL-1β levels showed a tendency of slight reduction in knock-out mice compared to WT controls (**Fig. S8c**).

**Figure 7:**
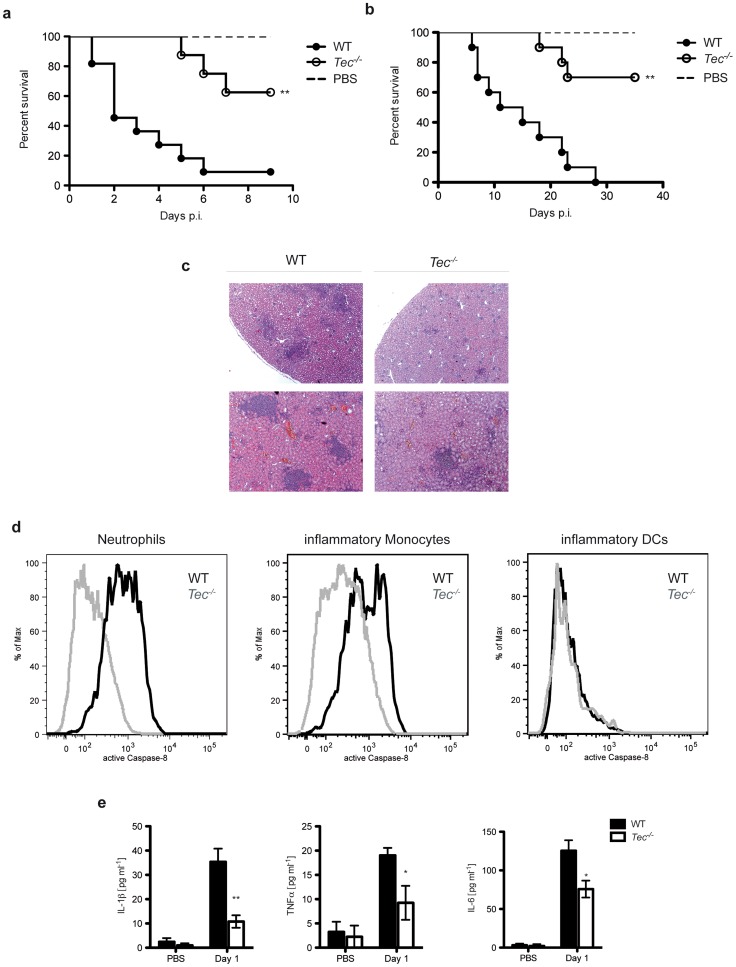
Tec-deficient mice are highly resistant to candidiasis. (**a**) Survival of mice after intraperitoneal infection (*i.p.*) with 5.10^7^ CfUs of *C. albicans*; for analysis of mouse survival curves Log-rank (Mantle-Cox) test was used. n = 12 per genotype. (**b**) Survival of mice after intravenous infection (*i.v.*) with 1.10^5^ CfUs of *C. albicans*; for analysis of mouse survival curves Log-rank (Mantle-Cox) test was used. n = 11 per genotype. (**c**) Histopathological analysis of mice after i.v. infection with 1.10^5^ CfUs of *C. albicans*; HE-Staining. (**d**) Intracellular staining of active caspase-8 in neutrophils (CD11b^+^Ly6C^+^Ly6G^+^), inflammatory monocytes (CD11b^+^Ly6C^+^Ly6G^−^) and inflammatory DCs (CD11b^+^CD11c^+^Ly6C^+^) in leukocytes isolated from kidneys after *i.v.* infection with 5.10^6^ CfUs of *C. albicans* for 24 h. (**e**) ELISA of respective cytokines in sera of mice after *i.v.* infection with 5.10^6^ CfUs of *C. albicans* for 24 h. Data are representative of two (**d,e**) independent experiments.

Finally, we addressed the question whether the observed reduction of the inflammatory response in Tec-deficient mice would impact to the survival of these animals. Remarkably, *Tec^−/−^* mice revealed significantly higher survival rates after intraperitoneal challenge with *Candida albicans* when compared to their wild-type littermates (**Fig. 7a** **; Fig. S8d**). In contrast, *Dectin-1^−/−^* mice displayed a similar survival compared to their wild-type controls (**Fig. S8e**).

Next, we wanted to test whether these results can be reconfirmed in an intravenous challenge model. Strikingly, Tec-deficient mice also showed a drastically reduced mortality after intravenous infection with *Candida albicans* (**Fig. 7b**). However, the apparent increased survival was not a consequence of lower fungal loads in kidneys of infected animals (**Fig. S8f**). Tec-deficient mice also revealed reduced immunopathology after 7 days of infection, as evident by the reduced size and number of inflammatory lesions in the kidneys (**Fig. 7c**). Next, we tested whether loss of Tec also would affect caspase-8 activity of leukocytes recruited in the kidneys of infected mice. In line, isolated leukocytes from intravenously infected Tec-deficient mice did reveal strongly reduced levels of active caspase-8 (**Fig. 7d**). Of note, as also seen after intraperitoneal infection, neutrophils displayed highest caspase-8 activity among recruited leukocytes. Finally, Tec^−/−^ mice revealed strongly reduced levels of mature IL-1β as well as other inflammatory cytokines in sera of infected mice (**Fig. 7e**)

**Figure 8:**
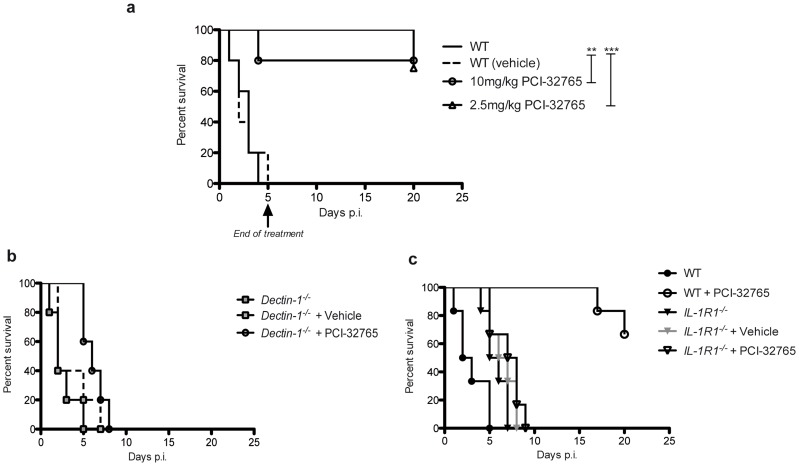
Chemical-genetic inhibition of Tec rescues mice from fatal fungal sepsis. (**a**) Survival of mice after intraperitoneal infection with 5.10^7^ CfUs of *C. albicans* and oral treatment with PCI-32765 with daily doses as indicated; treatment was stopped after 5 days; for analysis of mouse survival curves Log-rank (Mantle-Cox) test was used. n = 9 per group. (**b**) Survival of mice after intraperitoneal infection with 5.10^7^ CfUs of *C. albicans* and oral treatment with 5 mg/kg bodyweight PCI-32765 with daily doses; for analysis of mouse survival curves Log-rank (Mantle-Cox) test was used. n = 6 per group. (**c**) Survival of mice after intraperitoneal infection with 5.10^7^ CfUs of *C. albicans* and oral treatment with 5 mg/kg bodyweight PCI-32765 with daily doses; treatment was stopped after 9 days; for analysis of mouse survival curves Log-rank (Mantle-Cox) test was used. n = 6 per group.

Furthermore, to test whether chemical inhibition of Tec protects from candidemia-induced sepsis, we treated wild-type mice with the small molecule drug PCI-32765 (Ibrutinib), which inhibits Tec family kinases including Btk and Tec [33]. Strikingly, treatment of mice with PCI-32765 with two different doses for 5 consecutive days, dramatically increased survival of mice when compared to vehicle controls (**Fig. 8a**). Interestingly, dectin-1-deficient mice could not be rescued by Ibrutinib (**Fig. 8b**). Finally, to ultimately prove the involvement of Tec in the caspase-8-dependent maturation of IL-1β *in vivo*, we treated IL-1R1^−/−^ mice with Ibrutinib. Strikingly, in contrast to WT mice, IL-1R1-deficient mice were not rescued by chemical inhibition of Tec (**Fig. 8c**). Taken together, the data suggest novel antifungal strategies, by dampening the host hyper-inflammatory response following fungal infections. The results provide a proof-of-principle that Tec signaling in innate phagocytes tightly controls fungal immunity by controling the caspase-8-dependent maturation of IL-1β and the induction of hyperinflammation.

## Discussion

This work constitutes the first report for an innate-specific function of the intracellular non-receptor tyrosine kinase Tec in microbial pathogenesis. Tec is a novel signal transducer required for mounting the inflammatory response to microbial pathogens in innate immune cells. Tec family kinases are implicated in many signaling processes but evidence for a role in microbial virulence has been lacking. Notably, so far, only the Tec kinase family member Btk has been implicated in TLR-signaling [34], [35], in phagocytosis of microbes [15], [36] and recently in *Listeria monocytogenes* infections of macrophages [37]. In addition, Btk is involved in the phagocytosis of Candida [15]. Interestingly, this study reports quite opposite results for the role of Btk when compared to Tec in this study. Tec function does not require phagocytosis of fungal cells; Tec-deficient immune cells release significantly reduced levels of inflammatory cytokines in response to fungal challenge; and, strikingly, *Tec^−/−^* mice are hyper-resistant to fungal sepsis, whereas Btk mice are hypersusceptible. Hence, this study and the one from Ploegh and colleagues provide evidence for non-redundant functions of Tec family kinases in myeloid cells for the very first time. Btk might interact with a PRR during phagocytosis, while Tec may transduce signals in the sensing mode of the receptor. However, none of the mentioned studies identified in-depth mechanistic views as to how Btk might regulate host immune responses.

Here, we show that Tec-deficient macrophages display severe defects in the inflammatory host response upon fungal challenge as evident from decreased production of ROS and inflammatory cytokines. Interestingly, the MALT1 para-caspase activity, as well as caspase-8 activity itself contribute to NF-κB activation and transcription of inflammatory cytokines [38]–[40]. In line, our collective data confirm an essential role of caspase-8 during the dectin-1-dependent activation of NF-κB target genes. Therefore, it is likely that Tec may regulate transcription via these distinct pathways. Strikingly, however, only fungal PAMP-mimics such as zymosan or curdlan or live fungal pathogens can cause this phenotype in macrophages. In contrast, the LPS response is normal, suggesting that Tec is not involved in TLR4-signaling at all. Furthermore, our data demonstrate that Tec is required for caspase-8-dependent IL-1β processing in response to various species of clinical Candida isolates, but not upon challenge with either *Gram*-positive or *Gram*-negative bacterial pathogens. Hence, these data suggest a dedicated role for Tec in immune responses triggered by fungal but not bacterial pathogens, arguing for a TLR-independent role of Tec. This is consistent with the fact that fungal glucans are the main PAMPs activating the dectin-1-dependent host immune responses.

Activation of Tec requires both Src and Syk, while Tec activates PLCγ2. So far, however, PLCγ2 activation by Tec and Btk has only been reported to occur in lymphocytes, mast cells and osteoclasts under non-infectious conditions [22], [23], [41]. Notably, Syk signaling mediates CARD9-dependent responses [19]. Hence, impaired activation of PLCγ2 most likely decreases CARD9-dependent inflammasome formation in Tec-deficient cells. While several reports hint activation of Syk/Src in response to physiological as well as malignant stimuli [42], our “biochemical epistasis” nonetheless identifies Tec as a novel key component mediating Syk signaling during fungal infections. Because Syk is involved in both normal and malignant signaling [42], the extent to which Tec can simultaneously act in different pathways requires future attention. Although we identify dectin-1 as the major PRR activating Tec upon fungal challenge, one cannot exclude that Tec is activated by other Syk-dependent signaling pathways. Of note, dectin-1-deficient mice do not show increased survival during fungal sepsis as *Tec^−/−^* mice do, and dectin-1 mice cannot be rescued by chemical inhibition of Tec. While inconsistent at first sight, this is actually expected, since Tec modulates inflammatory immune responses, while dectin-1 represents an essential PRR in anti-fungal immunity. Moreover, *Dectin-1^−/−^* mice exhibit severe recruitment defects of immune cells after intraperitoneal challenge with *Candida albicans* [27], which is in sharp contrast to *Tec^−/−^* mice, where immune cell recruitment is unaffected.

Our results identify Tec kinase as the first known example of a Syk-dependent caspase-8-specific activator, since caspase-1 activation was entirely Tec-independent under the conditions used for macrophage infections. Interestingly, caspase-8 activity after fungal challenge was only transient in wild-type phagocytes, with a peak at 60 minutes, while caspase-1 activity seemed to be constant after fungal stimulation. Because both caspases mediate IL-1β processing and inflammation, one may speculate that pathogen-induced inflammation proceeds through a stepwise mechanism, so as to avoid hyper-inflammation. While early stimulation leads to the activation of caspase-8, persistent stimulation would then activate caspase-1 or both. The power of the inducing stimulus may therefore regulate which of the two caspase-containing inflammasomes get activated. Initial β-glucan sensing may activate caspase-8 rapidly, while persisting high glucan exposure, especially in hyphal tips of fungi, may lead to caspase-1 activation. This may equip innate immune cells with means to efficiently downregulate each step of the inflammatory response and avoid spill-over into apoptosis. The latter mechanism would ensure viability of phagocytes and help them to eliminate microbes more efficiently.

Interestingly, we show that MALT1 and ASC might be pre-associated, perhaps to constitute a pathogen-sensing complex, at least in murine BMMs. Indeed, there is evidence that caspase-8 and MALT1 might interact constantly without stimulus in human dendritic cells [11]. Such pre-activation complexes could provide a platform for the rapid induction of inflammation in different cell types and/or in species. Of note, we also show that the Tec-dependent activity of the caspase-8 inflammasome is phagocytosis-independent, indicating that dectin-1, besides its phagocytic mode for fungi, transmits signals outside phagosomes and without internalization through a signaling or sensing mode, has been proposed previously [11]. Therefore, it is tantalizing to speculate that Tec would only associate with dectin-1 in the sensing mode but not in the phagocytic mode. Nonetheless, a sensing mode of otherwise phagocytic PPRs might be extremely important for maintaining commensalism and tolerance, especially in the case of fungi colonizing mucosal layers that normally do not undergo phagocytosis. Beside the phagocytic mode of receptors to eliminate pathogens, the sensing mode might provide an immunological-relay: if the pathogenic signal is below a certain threshold, cells will not activate innate or adaptive immunity, leading to the formation of tolerance and commensalism between the host and a microbe. However, if a stimulus is strong enough and persisting, cells will induce an immune response, including the activation and recruitment of migratory innate as well as adaptive immune cells, as for instance observed during the commensal to pathogen switch in the intestine [43]. As many innate cells are strongly inflammatory (neutrophils, monocytes, DCs and tipDCs), this may explain the promotion of sepsis and the often observed lethal side-effects for the host due to oxidative tissue and organ damage [44], [45]

Importantly, we use two distinct mouse infection models to verify the inflammatory function of Tec *in vivo*: the intraperitoneal infection model for local inflammation and acute sepsis, and the normally used intravenous challenge model leading to disseminated systemic candidiasis. Our data clearly confirm the role of Tec in the caspase-8-dependent maturation of IL-1β including the *in vivo* role of Tec in the induction of a hyperinflammatory antifungal response. In addition, the results imply an innate-specific cellular rather than a systemic function of Tec, since the recruitment and composition of immune cells is not affected by the lack of Tec. Interestingly enough, immune cell functions for Tec have only been reported for T cells and mast cells so far [23], [46] but not for innate phagocytes. Furthermore, we identify neutrophils as the major source of active caspase-8 after intraperitoneal as well as after intravenous challenge. Of note, this is completely in line with the notion that neutrophils are the major source of inflammation-driven tissue destruction and immunopathology in systemic candidiasis [44], [45]. Notably, we could not use caspase-8-deficient mice to confirm the role of Tec in caspase-8 inflammasome signaling, since caspase-8 deficiency causes embryonic lethality [47]. Moreover, we believe that Tec constitutes a molecular fine-tuner of pathogenic inflammatory signalling. Thus, even a conditional loss or chemical inhibition of caspase-8 may cause pleiotropic effects, thereby masking the observed phenotypes. Notably, caspase-8 plays an important role during the downregulation of inflammatory immune responses by actively inducing apoptosis in immune cells [48]. Hence, a complete block of caspase-8 during an immune response *in vivo* is likely to result in hyper-inflammation and increased induction of sepsis.

Importantly, our data not only discover Tec as a novel major fungal immune response regulator, they also show a therapeutic potential of Tec. The chemical inhibition of Tec kinase signaling by PCI-32756 has only been used for treatment of cancer so far [49]. However, Tec inhibitors for treatment of infectious diseases could represent a novel therapeutic strategy as it targets the host response rather than the pathogen. Hence, this work also provides compelling evidence in support of for new strategies in antifungal drug discovery. Till today, only few classes of antifungals are available yet all drugs inhibit aspects of pathogen growth [50]. Therefore, attenuating hyper-inflammation and sepsis in patients could provide a therapeutic option, especially when considering that patients and animals with disseminated fungal infections die from a slow progressing sepsis [51], [52]. Hence, Tec may be one of the first promising host-targets to combat fungal infections, at the same time perhaps diminishing the toxic side effects seen for existing antifungal drugs, especially for critically ill patients. Interestingly enough, to date, human mutations in TEC associated with decreased susceptibility to microbial infections have not been reported. The most obvious and tantalizing explanation would be that this patient cohort may cope much better with invasive fungal infections, since attenuated sepsis development and increased tolerance to invasive fungal infections would constitute clear survival benefits.

## Methods

### Ethics statement

All animal experiments were evaluated by the ethics committee of the Medical University of Vienna and approved by the Federal Ministry for Science and Research, Vienna, Austria (GZ: BMWF-68.20n5/231-II/3b/2011).

### Mice infections and fungal strains

*Tec^−/−^* [16], *Dectin-1^−/−^ (Clec7a^−/−^)* [53], *Dectin-2^−/−^ (Clec4n^−/−^)* [54], *TLR2/4^−/−^* [55], *ΦCγP^−/−^* [56] and *IL-1R1^−/−^* [57] mice have been described. Corresponding WT (C57BL/6) and knockout mice were maintained in the animal facility of the Medical University of Vienna/Max F. Perutz Laboratories. Mice (8–10 weeks old) were infected with 1× resp. 5×10^7^ colony-forming units (CFU) of *C. albicans* (strain SC5314) or 3% brewer thioglykolate medium (Sigma) intraperitoneally (*i.p.*) or 1×10^5^ CFUs of Ca intravenously. PCI-32765 (Selleckchem) was administered by oral gavage daily with indicated concentrations.

For the quantification of cellular infiltrates as well as cytokines, mice were sacrificed and a peritoneal lavage (PBS) was performed. Cellular infiltrates were quantified and analyzed, as indicated, via FACS. Neutrophils were isolated from tibias and femurs of mice using a Percoll-gradient (GE Healthcare).

Fungal strains used in this study included *C. albicans* strain SC5314 and clinical isolates of *Candida albicans*, *Candida glabrata*, *Candida krusei, Candida lusitaniae* as well as *Cryptococcus neoformans*. Bacterial strains in this study included *Pseudomonas aeruginosa* strain Pao1 *Listeria monocytogenes* strain EGD, *Staphylococcus aureus* strain COL and *Streptococcus pyogenes* strain SF370.

### BMM culture, stimulation, inhibition and RNA-mediated interference

BMMs were derived from bone marrow of WT or respective knockout mice as described [58] and stimulated with pathogens (see above) zymosan (Sigma-Aldrich), ultrapure LPS, Curdlan, Pam_3_CSK_4_ (all Invivogen). Where indicated, cells were pretreated 30 min prior stimulation with the Src kinase inhibitor PP2 (Selleckchem) or its inactive analog PP3 (Calbiochem), the Syk kinase inhibitor R406 (Selleckchem), caspase-1 inhibitor z-YVAD-fmk, caspase-8 inhibitor z-IETD-fmk (both BioVision), anti-Dectin 1 antibody, anti-Mincle antibody, anti-Trem1 antibody (all Invivogen), anti-Dectin 2 antibody (R&D Systems), Laminarin (Invivogen), Cytochalasin D, Bafilomycin A_1_, Dynasore or Puromycin (all Sigma-Aldrich).

For RNA-mediated interference, 8-day-old BMMs were transfected with 25 nm siRNA through the use of the transfection reagent DF4 (Dharmacon). 72 h after transfection cells were used for experiments. The following SMARTpool siGENOME siRNAs were used: caspase-8 (M-043044-01), MALT1 (M-051221-01), CARD9 (M-045760-01), Bcl-10 (M-043902-00), caspase-1 (M-043902-00), ASC (M-051439-01) and nontargeting siRNA, as a control (D-001210-01) (all from Dharmacon). Silencing of expression was verified by real-time PCR and immunoblot.

### Reactive oxygen species measurements & caspase activity assays

Production of reactive oxygen species (ROS) was measured in real-time over 120 minutes exactly as described earlier [58]. BMMs were stimulated as indicated and activity of caspase-3, caspase-7, caspase-8 was measured using Caspase-Glo® Assay (Promega) according to manufacturer's recommendations. Activity of caspase-1 was determined by Caspase-1 Colorimetric Assay Kit (BioVision). Whereas processing of caspase-1, caspase-3, caspase-8 and caspase-9 was detected by immunoblot. Multiplicity of infection was 2∶1 (2 fungi per one immune cell).

### *In vitro* kinase assays

Recombinant active Syk-Kinase was obtained from Millipore (Billerica, USA), active Tec-Kinase was bought from Sigma-Aldrich. In brief, Tec, resp. PLCγ2, was immunoprecipitated from unstimulated BMMs using Dynabeads (Life Technologies). 1 µg bead-bound Tec, resp. PLCγ2, was then incubated for 30 min at 30°C with 70 ng active Syk or Tec in 50 µl kinase buffer consisting of 25 mM HEPES, pH 7.4, 25 mM MgCl_2_, 25 mM β-glycerophosphate, 0.1 mM Na-Vanadate supplemented with 200 nM ATP (Sigma). Reaction was terminated with Laemmli buffer, samples were boiled and subjected to SDS-PAGE and immunoblot analysis.

### Cytokine expression and release

mRNA was isolated with SV-Total RNA Isolation System (Promega), cDNA was generated using the Reverse Transcription System (Promega) and qPCR was performed with KAPA SYBR FAST Universal (Peqlab) as described in [58]. For primer sequences see Table S1. Concentrations of TNFα, pro-IL-1β, IL-1β and IL-12 released into cell culture supernatants from unstimulated and stimulated BMMs, as well as from peritoneal lavage or present in cell-lysates were analyzed with plate-bound ELISA kits (ELISA Ready-SET-Go!, all eBioscience).

### Immunoblotting and antibodies

Protein immunoblots were performed as described before [58] using primary antibodies against phospho-Src (Tyr416), phospho-Syk (Tyr525/526), phospho-Tyrosine (P-Tyr-100), phospho-PLCγ2 (Tyr759), phospho-PKCδ (Tyr311), phospho-IκBα (Ser32/36), phospho-IKKα/β (Ser176/180), phospho-p42/44 MAPK (Thr202/Tyr204), phospho-NF-κB p65 (Ser356), phopsho-c-Raf (Ser338), IκBα, Caspase-8, Bcl10, MALT1, Caspase-3, Caspase-9 and PARP (all Cell Signaling Technology), p38, Card9 (both Santa Cruz Biotechnology), Tec (Upstate Millipore) and Caspase-1 (Invitrogen). HRP-coupled anti-mouse or anti-rabbit secondary antibodies (both Cell Signaling Technology) were used.

For coimmunoprecipitation experiments, BMMs were stimulated with *Candida albicans* as indicated and lysed in NP-40-containing buffer (Cell Signaling Technology). Protein complexes were precipitated with indicated antibodies using the Immunoprecipitation Kit – Dynabeads Protein G (Life Technologies) according to manufacturer's recommendations. Eluted complexes were then subjected to protein immunoblotting and detected by use of Clean-Blot IP Detection Reagent (Thermo Scientific) as secondary antibody.

### Enrichment of phosphorylated proteins

Phosphorylated proteins were enriched from cell lysates with a Pro-Q Diamond Phosphoprotein Enrichment Kit (Invitrogen) according to the manufacturer's protocol. Briefly, cells were lysed in lysis buffer supplemented with inhibitors of endonuclease and proteinase. Using non-denaturing conditions, lysates were loaded on the columns, concentrated and finally precipitated with methanol and chloroform. The resulting pellet was dried and resolubilized with SDS sample buffer. Tec or phosphorylated Tec, as well as a loading control were detected by immunoblot analysis.

### Generation of *efg1* and *efg1 cph1 Candida albicans* mutant strains

All *C. albicans* strains were derived from the MTLa/α clinical isolate SC5314. Homozygous deletion gene was achieved using the SAT1-flipper technique [59]. The *efg1* mutant was described earlier [60]. For *efg1*Δ/Δ *cph1*Δ/Δ double mutants, a suitable *cph1* deletion cassette was constructed using the fusion PCR strategy [61].

### Data analysis and statistics

Data were analyzed using commercial software Prism 5.0a (GraphPad Software). Data are represented as mean ± SD. For analysis of mouse survival curves Log-rank (Mantle-Cox) test was used. For statistical analysis unpaired t-tests with 95% confidence intervals were used. P values such as * p-value<0,05; ** p-value<0,01; *** p-value<0,001 were considered significant.

## Supporting Information

Figure S1 (**a**) Cell numbers of *in vitro* differentiated BMMs according to CD11b^+^F4/80^+^ cells assessed by fluorescence activated cell sorting (FACS). (**b**) Immunoblot analysis of Tec and qPCR analysis of Tec expression after stimulating BMMs with *C. albicans* for 120 min; results are normalized to GAPDH (glyceraldehyde phosphate dehydrogenase). (**c**) ELISA of indicated cytokines in supernatants of BMMs after *C. albicans* (Ca) stimulation with different multiplicities of infection (MOI; fungi:BMM) or unstimulated (Unstimul). (**c**) Rate of phagocytosis after 45 Min of incubation with *C. albicans* (Ca). (**d**) ELISA of TNFα in supernatants of BMMs after stimulation with increasing doses of lipopolysaccharide (LPS), zymosan or curdlan or Pam_3_CSK_4_ (1 µg/ml) (**e**) Immunoblot analysis of p-ERK and p-p38 activation in the time course of *C. albicans* infection in BMMs. Data are representative of at least seven (**a**), three (**b,c**) or two (**d**,**e**) independent experiments. Mean and SD are shown.
(TIFF)

Figure S2 (**a**) Caspase-1 activity after 60 Min of stimulation with *C. albicans* (Ca) or with dimethylsulfoxide (DMSO), Casp1 inhibitor (Casp1 Inh; 5 mM) or Casp8 inhibitor (Casp8 Inh; 5 mM) and Ca; absorbence of unstimulated cells, cells with respective inhibitor, cells with DMSO or *C. albicans* only was subtracted. (**b**) Caspase-8 activity after 60 Min of stimulation with *C. albicans* (Ca) or with dimethylsulfoxide (DMSO), Casp1 inhibitor (Casp1 Inh; 5 mM) or Casp8 inhibitor (Casp8 Inh; 5 mM) and Ca; chemiluminenscence of unstimulated cells, cells with respective inhibitor, cells with DMSO and *C. albicans* only was subtracted. (**c**) Caspase-3/7 activity over the course of infection with *C. albicans*; chemiluminenscence of unstimulated cells and *C. albicans* only was subtracted (full line); Caspase3/7 activity of BMMs stimulated with 5 mM puromycin (dashed line) (**d**) Immunoblot analysis of full-length and active/cleaved subunits of poly ADP ribose polymerase (PARP), caspase-3 and caspase-9 after stimulation with C. albicans or puromycin (Puro; 5 mM) for 120 Min. Data are representative of at least three (**a–c**) or two (**d**) independent experiments. Mean and SD are shown (**a–c**).
(TIFF)

Figure S3 (**a**) qPCR analysis of indicated targets without (basal expression) or after stimulation with *C. albicans* (Ca) for 120 Min; results are normalized to those of GAPDH. (**b**) Immunoblot analysis of CARD9, Bcl-10, MALT1, ASC and caspase-8 of cells left untreated (untr), knockdown of a non-target (nTG; 25 nM) or respective siRNA knock down (25 nM) after 48 hrs of incubation. (**c**) qPCR analysis of indicated targets of cells left untreated, knockdown of a non-target (nTG; 25 nM) or respective siRNA knock down (25 nM) after 72 hrs of incubation. Data are representative of at least three (**a,c**) or two (**b**) independent experiments. Mean and SD are shown (**a,c**).
(TIFF)

Figure S4 (**a**) ELISA of IL-1β in supernatants of BMMs after stimulation with *C. albicans* only (Ca) or with dimethylsulfoxide (DMSO), CytochalasinD (CytoD; 2 µM) or Dynasore (80 µM) and Ca or left untreated (-). (**b**) ELISA of IL-1β in supernatants of BMMs after stimulation with *C. albicans* only (Ca) or with dimethylsulfoxide (DMSO) or BafilomycinA_1_ (BafiloA_1_; 30 nM) and Ca or left untreated (-). (**c**) ELISA of IL-1β in supernatants of BMMs after stimulation with *C. albicans* only (Ca) or with dimethylsulfoxide (DMSO), Syk Inhibitor R406 (3 µM), Src Inhibitor PP2 (5 µM) or the non-functional analogon PP3 (5 µM) and Ca or left untreated (-). (**d**) ELISA of pro-IL-1β in cell lysates of BMMs after stimulation with *C. albicans* only (Ca) or with dimethylsulfoxide (DMSO), CytochalasinD (CytoD; 2 µM), Dynasore (80 µM), BafilomycinA_1_ (BafiloA_1_; 30 nM), Syk Inhibitor R406 (3 µM), Src Inhibitor PP2 (5 µM) or the non-functional analogon PP3 (5 µM) and Ca or left untreated (-). (**e**) ELISA of pro-IL-1β in cell lysates and IL-1β in supernatants of BMMs after stimulation with *C. albicans* in BMMs of indicated genotype or left unstimulated (-). (**f**) ELISA of pro-IL-1β in cell lysates and IL-1β in supernatants of BMMs after stimulation with *C. albicans* in WT BMMs blocked with indicated antibodies (all 10 µg/ml) and respective isotype control (10 µg/ml) or left unstimulated (-). Data are representative of at least two three (**a**–**f**) independent experiments. Mean and SD are shown.
(TIFF)

Figure S5 (**a**–**b**) Immunoblot analysis of CARD9, Bcl-10, MALT1, ASC and caspase-8 (Casp8) after immunoprecipitation (IP) with antibodies against CARD9 (**a**), MALT1, ASC and caspase-8 (**b**) from whole-cell lysates of BMMs left unstimulated (-) or stimulated with *C. albicans* for 60 Min. Data are representative of two independent experiments for each IP.
(TIFF)

Figure S6 (**a**) Microscopy of Candida mutants under hyphal-inducing conditions. Upper panel: cells were grown on YPD agar +10% FCS at 37°C for 3 days; scale bar: 1 mm. Middle panel: cells were grown in liquid YPD +10% FCS at 37°C for 1 hour; DIC = 100×; scale bar: 20 µm. Lower panel: same as middle panel; cells were stained with 10 µM Calcofluor White. (**b**) ELISA of pro-IL-1β in cell lysates of BMMs after stimulation with *Candida albicans* (Ca), Curdlan (200 µg/ml), heat-killed Ca, *efg1*Δ/Δ or *efg1*Δ/Δ *cph1*Δ/Δ Ca-mutants. Data are representative of at least two independent experiments (**a,b**).
(TIFF)

Figure S7 Caspase-8 activity after 60 Min and ELISA of IL-1β in supernatants of BMMs after 4 h of stimulation with respective clinical isolate. Clinical isolates of *Candida albicans* (**a**), *Candida glabrata* (**b**), *Candida krusei* (**c**), *Candida lusitaniae* (**d**) and *Cryptococcus neoformans* (**e**) were used. Data are representative of at least three independent experiments. Mean and SD are shown.
(TIFF)

Figure S8 (**a**) Cell numbers of recruited peritoneal cells upon intraperitoneal infection (*i.p.*) with 5.10^6^ CfUs of *C. albicans* after indicated time of infection or cells from uninfected mice; Cell type was assessed by FACS; Neutrophils: CD11b^+^Ly6G^+^F4/80^−^, Macrophage: CD11b^+^F4/80^+^; n = 3 per genotype and time point. (**b**) Cell numbers of recruited peritoneal cells upon intraperitoneal infection (*i.p.*) with 3% brewer thioglykolate medium for 4 days or cells from uninfected mice; Cell type was assessed by FACS; peritoneal macrophages: CD11b^+^F4/80^+^; n = 4 per genotype and time point. (**c**) ELISA of pro-IL-1β in cell lysates of peritoneal cells after indicated time of infections. (**d**) Survival of mice after intraperitoneal infection (*i.p.*) with 1.10^7^ CfUs of *C. albicans*. n = 6 per genotype (**e**) Survival of mice after intraperitoneal infection (*i.p.*) with 5.10^7^ CfUs of *C. albicans*.; for analysis of mouse survival curves Log-rank (Mantle-Cox) test was used. n = 6 per genotype. (**f**) Fungal loads in kidneys of mice after intravenous infection (*i.v.*) with 1.10^5^ CfUs of *C. albicans* 7 days post infection; n = 6 per genotype. Data are representative of at least three (**a**–**c**) independent experiments. Mean and SD are shown (**a**–**c**).
(TIFF)

Figure S9 (**a**) Model.
(TIFF)

Table S1 Sequences of real-time primers used in this study.
(TIFF)
