# Supplementary figures and images for: The Non-receptor Tyrosine Kinase Tec Controls Assembly and Activity of the Noncanonical Caspase-8 Inflammasome
Source: PLoS Pathog. 2014 Dec 4;10(12):e1004525. doi: 10.1371/journal.ppat.1004525 (PMC4256681; doi:10.1371/journal.ppat.1004525)

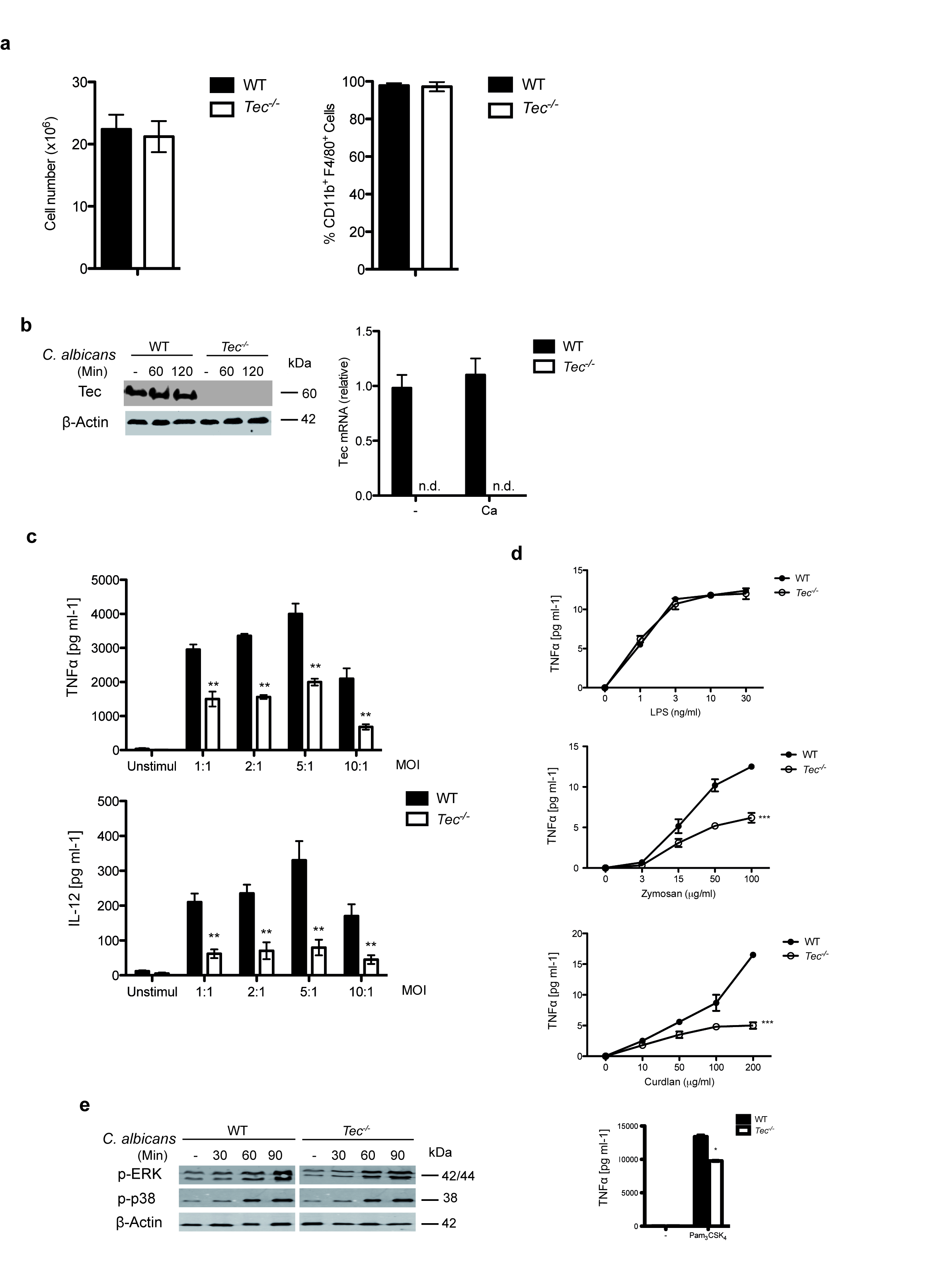

Supplement: Figure S1 — (a) Cell numbers of in vitro differentiated BMMs according to CD11b+F4/80+ cells assessed by fluorescence activated cell sorting (FACS). (b) Immunoblot analysis of Tec and qPCR analysis of Tec expression after stimulating BMMs with C. albicans for 120 min; results are normalized to GAPDH (glyceraldehyde phosphate dehydrogenase). (c) ELISA of indicated cytokines in supernatants of BMMs after C. albicans (Ca) stimulation with different multiplicities of infection (MOI; fungi:BMM) or unstimulated (Unstimul). (c) Rate of phagocytosis after 45 Min of incubation with C. albicans (Ca). (d) ELISA of TNFα in supernatants of BMMs after stimulation with increasing doses of lipopolysaccharide (LPS), zymosan or curdlan or Pam3CSK4 (1 µg/ml) (e) Immunoblot analysis of p-ERK and p-p38 activation in the time course of C. albicans infection in BMMs. Data are representative of at least seven (a), three (b,c) or two (d,e) independent experiments. Mean and SD are shown. (TIFF) [file ppat.1004525.s001.tiff]

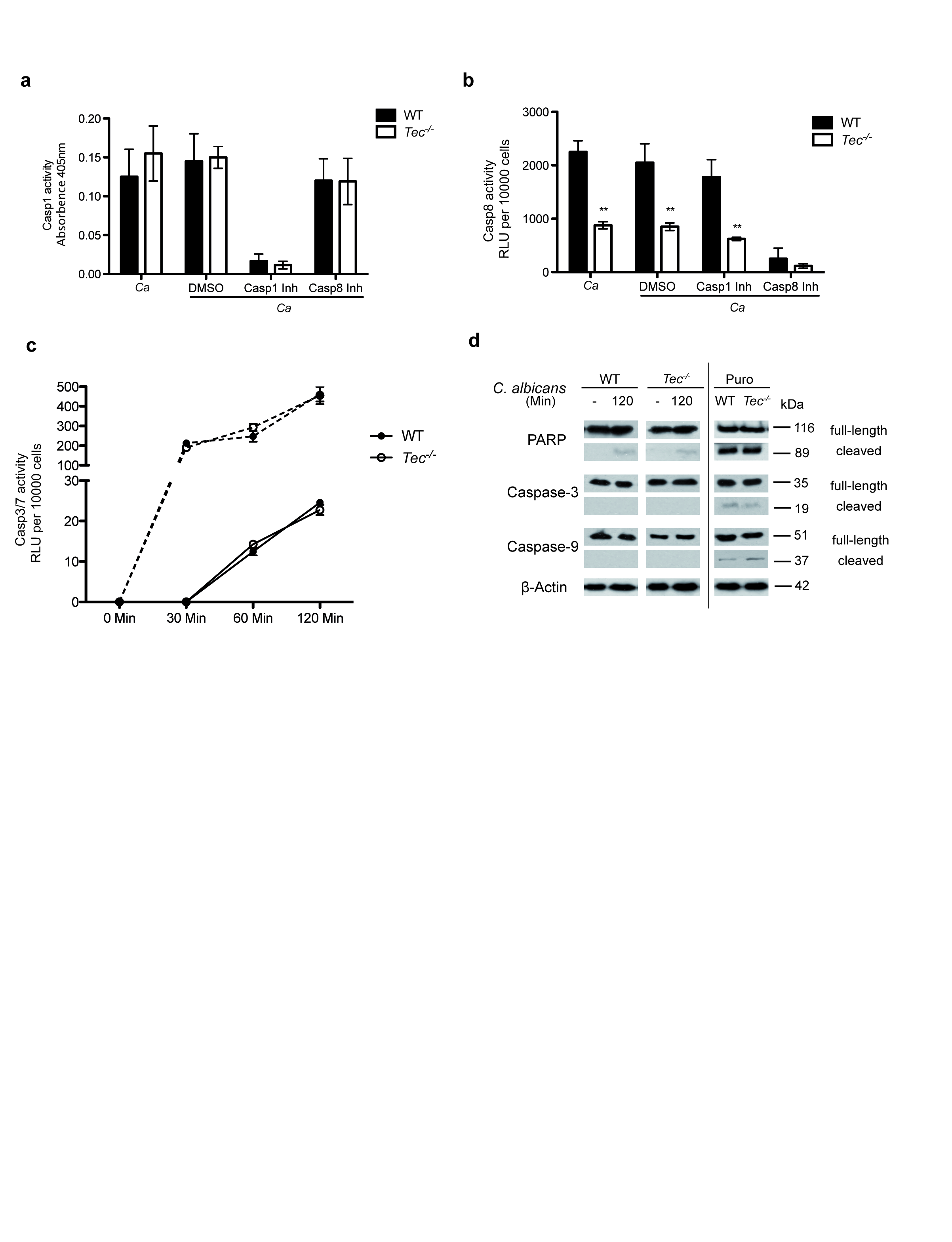

Supplement: Figure S2 — (a) Caspase-1 activity after 60 Min of stimulation with C. albicans (Ca) or with dimethylsulfoxide (DMSO), Casp1 inhibitor (Casp1 Inh; 5 mM) or Casp8 inhibitor (Casp8 Inh; 5 mM) and Ca; absorbence of unstimulated cells, cells with respective inhibitor, cells with DMSO or C. albicans only was subtracted. (b) Caspase-8 activity after 60 Min of stimulation with C. albicans (Ca) or with dimethylsulfoxide (DMSO), Casp1 inhibitor (Casp1 Inh; 5 mM) or Casp8 inhibitor (Casp8 Inh; 5 mM) and Ca; chemiluminenscence of unstimulated cells, cells with respective inhibitor, cells with DMSO and C. albicans only was subtracted. (c) Caspase-3/7 activity over the course of infection with C. albicans; chemiluminenscence of unstimulated cells and C. albicans only was subtracted (full line); Caspase3/7 activity of BMMs stimulated with 5 mM puromycin (dashed line) (d) Immunoblot analysis of full-length and active/cleaved subunits of poly ADP ribose polymerase (PARP), caspase-3 and caspase-9 after stimulation with C. albicans or puromycin (Puro; 5 mM) for 120 Min. Data are representative of at least three (a–c) or two (d) independent experiments. Mean and SD are shown (a–c). (TIFF) [file ppat.1004525.s002.tiff]

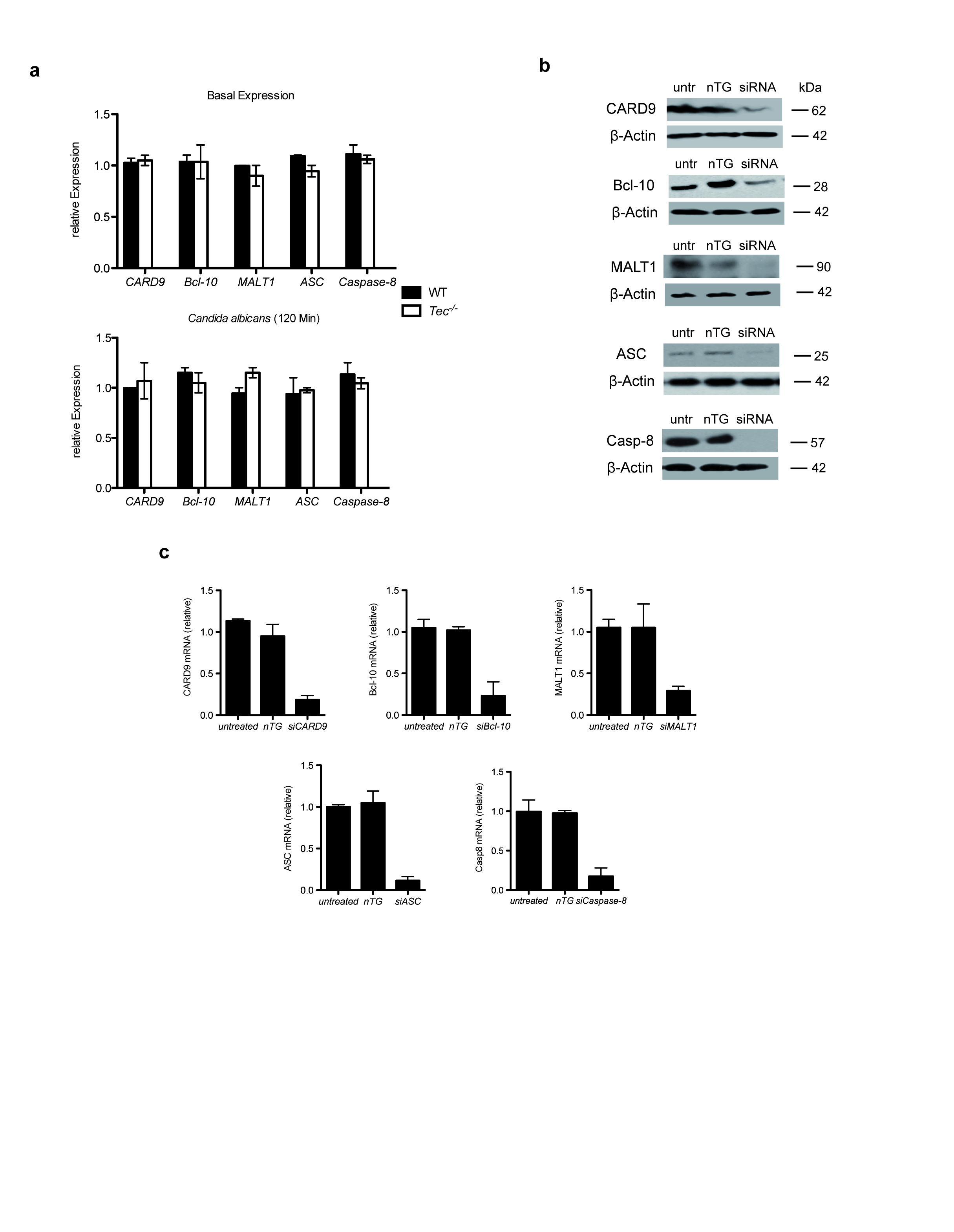

Supplement: Figure S3 — (a) qPCR analysis of indicated targets without (basal expression) or after stimulation with C. albicans (Ca) for 120 Min; results are normalized to those of GAPDH. (b) Immunoblot analysis of CARD9, Bcl-10, MALT1, ASC and caspase-8 of cells left untreated (untr), knockdown of a non-target (nTG; 25 nM) or respective siRNA knock down (25 nM) after 48 hrs of incubation. (c) qPCR analysis of indicated targets of cells left untreated, knockdown of a non-target (nTG; 25 nM) or respective siRNA knock down (25 nM) after 72 hrs of incubation. Data are representative of at least three (a,c) or two (b) independent experiments. Mean and SD are shown (a,c). (TIFF) [file ppat.1004525.s003.tiff]

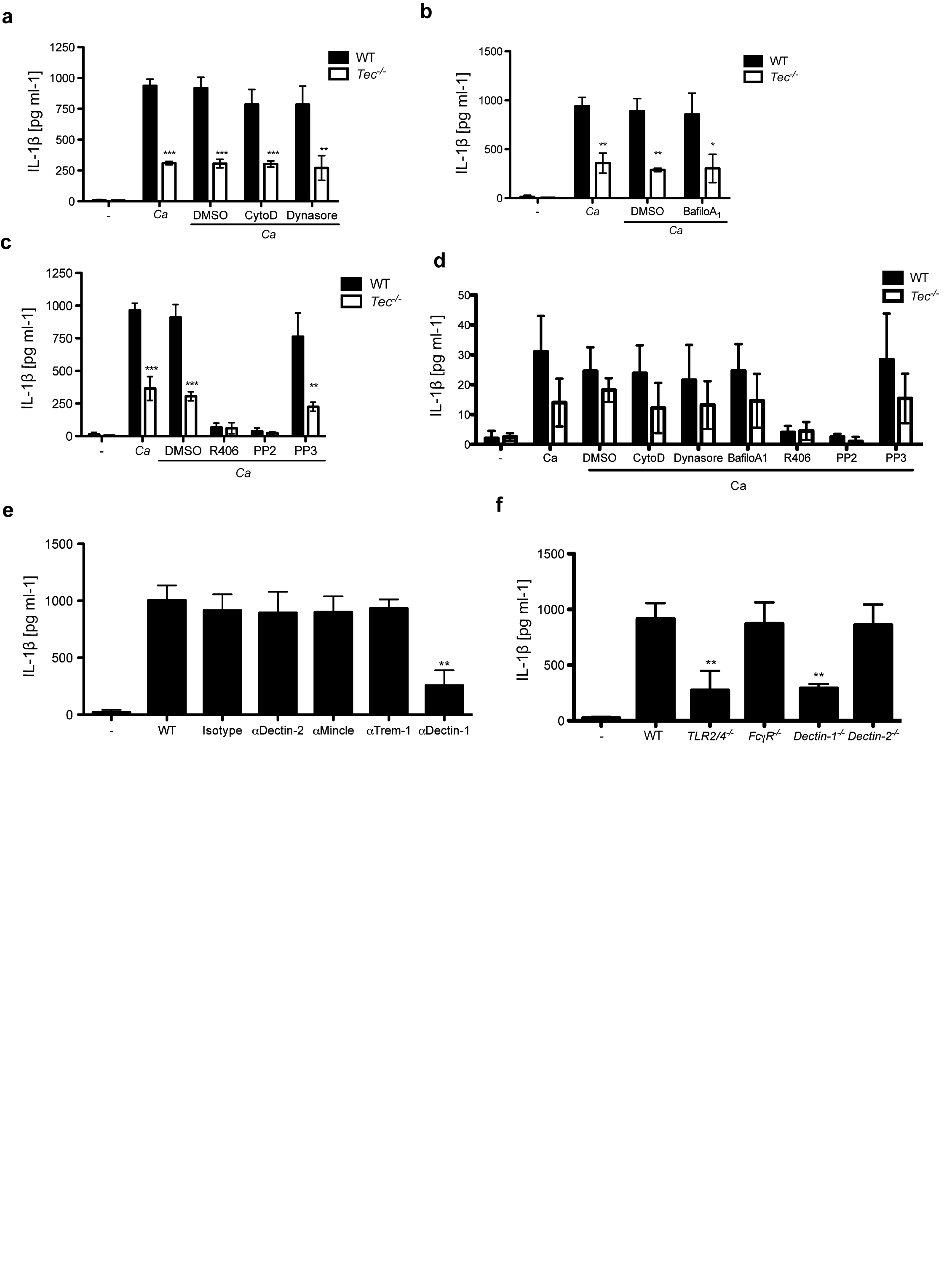

Supplement: Figure S4 — (a) ELISA of IL-1β in supernatants of BMMs after stimulation with C. albicans only (Ca) or with dimethylsulfoxide (DMSO), CytochalasinD (CytoD; 2 µM) or Dynasore (80 µM) and Ca or left untreated (-). (b) ELISA of IL-1β in supernatants of BMMs after stimulation with C. albicans only (Ca) or with dimethylsulfoxide (DMSO) or BafilomycinA1 (BafiloA1; 30 nM) and Ca or left untreated (-). (c) ELISA of IL-1β in supernatants of BMMs after stimulation with C. albicans only (Ca) or with dimethylsulfoxide (DMSO), Syk Inhibitor R406 (3 µM), Src Inhibitor PP2 (5 µM) or the non-functional analogon PP3 (5 µM) and Ca or left untreated (-). (d) ELISA of pro-IL-1β in cell lysates of BMMs after stimulation with C. albicans only (Ca) or with dimethylsulfoxide (DMSO), CytochalasinD (CytoD; 2 µM), Dynasore (80 µM), BafilomycinA1 (BafiloA1; 30 nM), Syk Inhibitor R406 (3 µM), Src Inhibitor PP2 (5 µM) or the non-functional analogon PP3 (5 µM) and Ca or left untreated (-). (e) ELISA of pro-IL-1β in cell lysates and IL-1β in supernatants of BMMs after stimulation with C. albicans in BMMs of indicated genotype or left unstimulated (-). (f) ELISA of pro-IL-1β in cell lysates and IL-1β in supernatants of BMMs after stimulation with C. albicans in WT BMMs blocked with indicated antibodies (all 10 µg/ml) and respective isotype control (10 µg/ml) or left unstimulated (-). Data are representative of at least two three (a–f) independent experiments. Mean and SD are shown. (TIFF) [file ppat.1004525.s004.tiff]

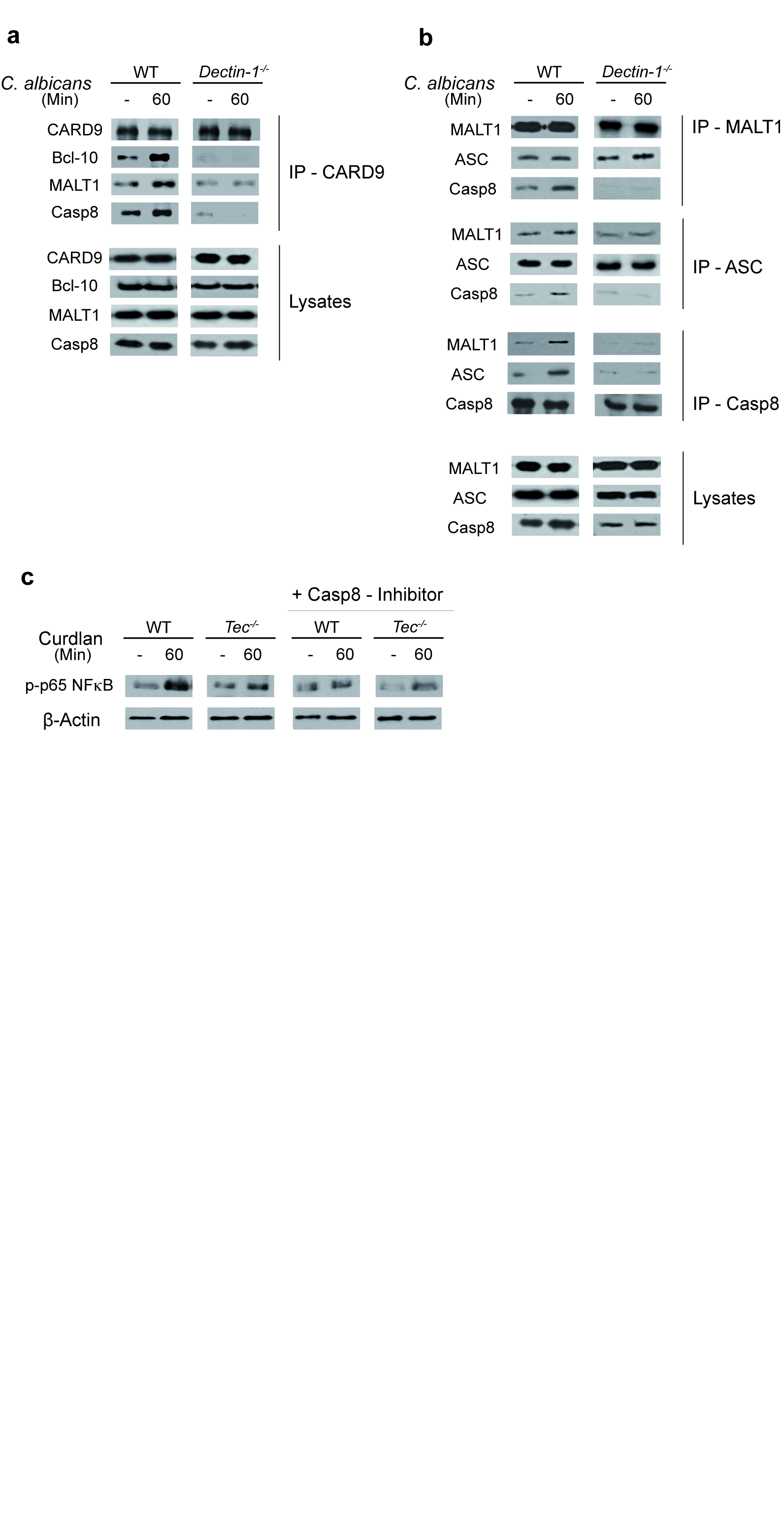

Supplement: Figure S5 — (a–b) Immunoblot analysis of CARD9, Bcl-10, MALT1, ASC and caspase-8 (Casp8) after immunoprecipitation (IP) with antibodies against CARD9 (a), MALT1, ASC and caspase-8 (b) from whole-cell lysates of BMMs left unstimulated (-) or stimulated with C. albicans for 60 Min. Data are representative of two independent experiments for each IP. (TIFF) [file ppat.1004525.s005.tiff]

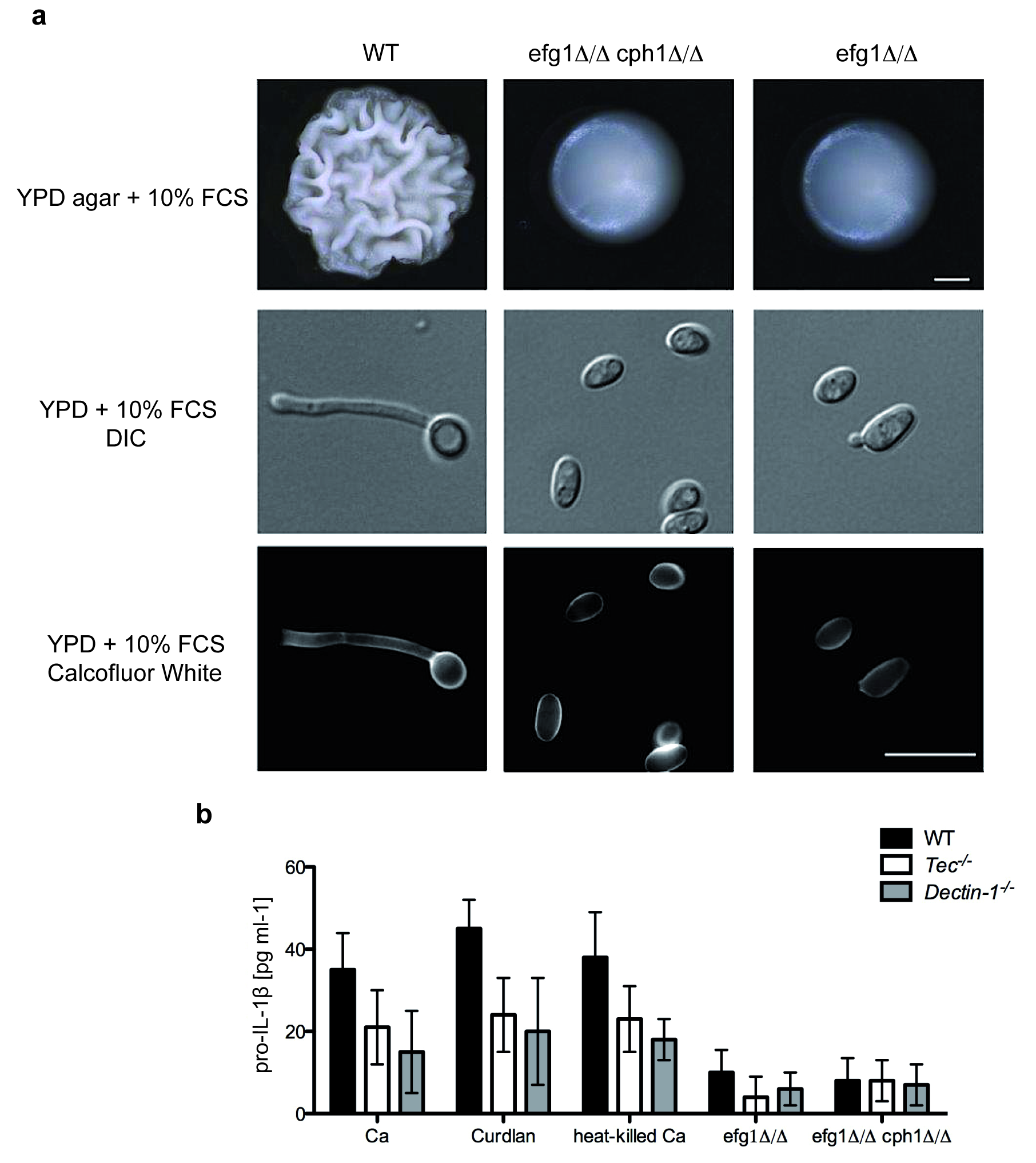

Supplement: Figure S6 — (a) Microscopy of Candida mutants under hyphal-inducing conditions. Upper panel: cells were grown on YPD agar +10% FCS at 37°C for 3 days; scale bar: 1 mm. Middle panel: cells were grown in liquid YPD +10% FCS at 37°C for 1 hour; DIC = 100×; scale bar: 20 µm. Lower panel: same as middle panel; cells were stained with 10 µM Calcofluor White. (b) ELISA of pro-IL-1β in cell lysates of BMMs after stimulation with Candida albicans (Ca), Curdlan (200 µg/ml), heat-killed Ca, efg1Δ/Δ or efg1Δ/Δ cph1Δ/Δ Ca-mutants. Data are representative of at least two independent experiments (a,b). (TIFF) [file ppat.1004525.s006.tiff]

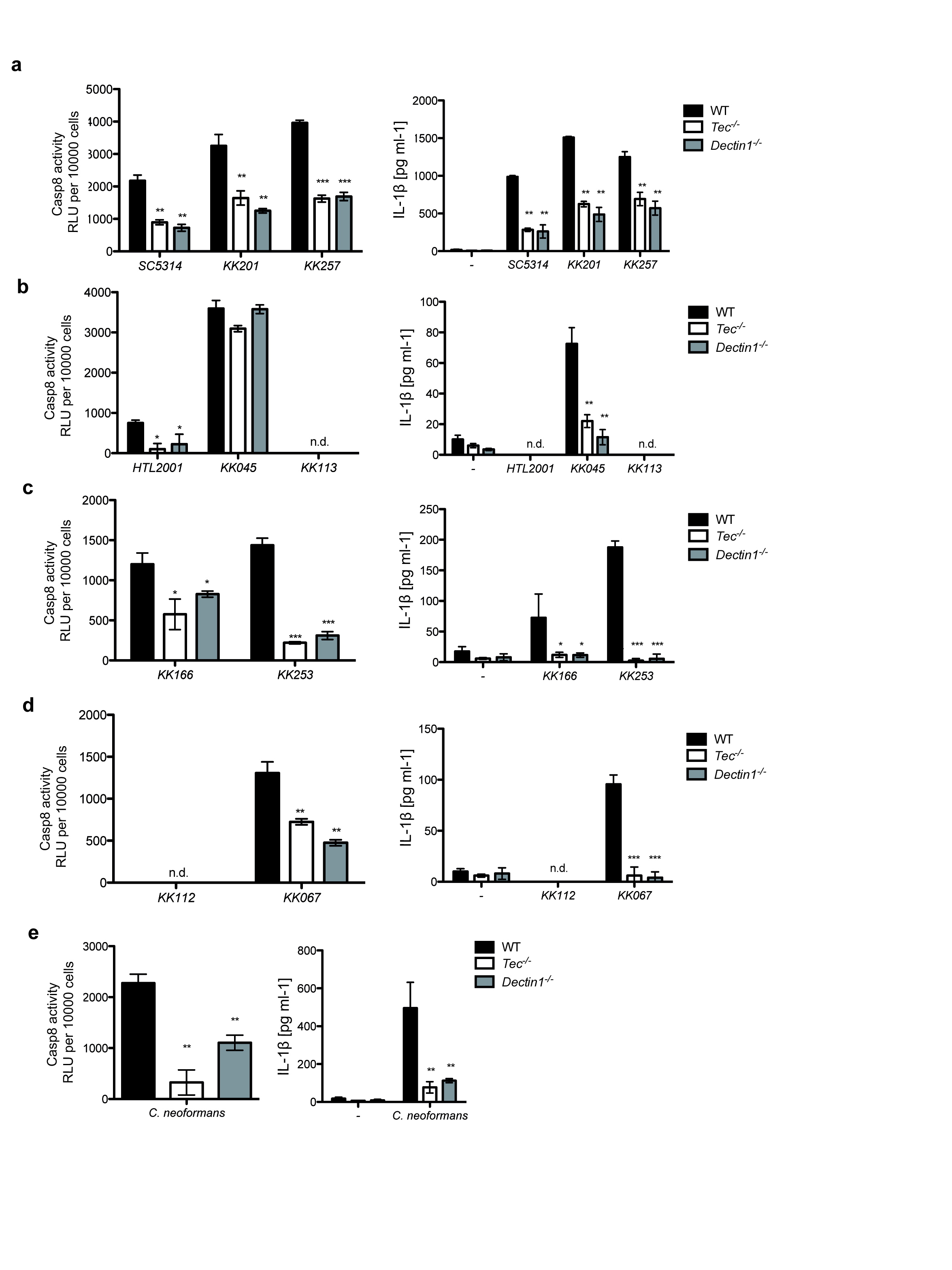

Supplement: Figure S7 — Caspase-8 activity after 60 Min and ELISA of IL-1β in supernatants of BMMs after 4 h of stimulation with respective clinical isolate. Clinical isolates of Candida albicans (a), Candida glabrata (b), Candida krusei (c), Candida lusitaniae (d) and Cryptococcus neoformans (e) were used. Data are representative of at least three independent experiments. Mean and SD are shown. (TIFF) [file ppat.1004525.s007.tiff]

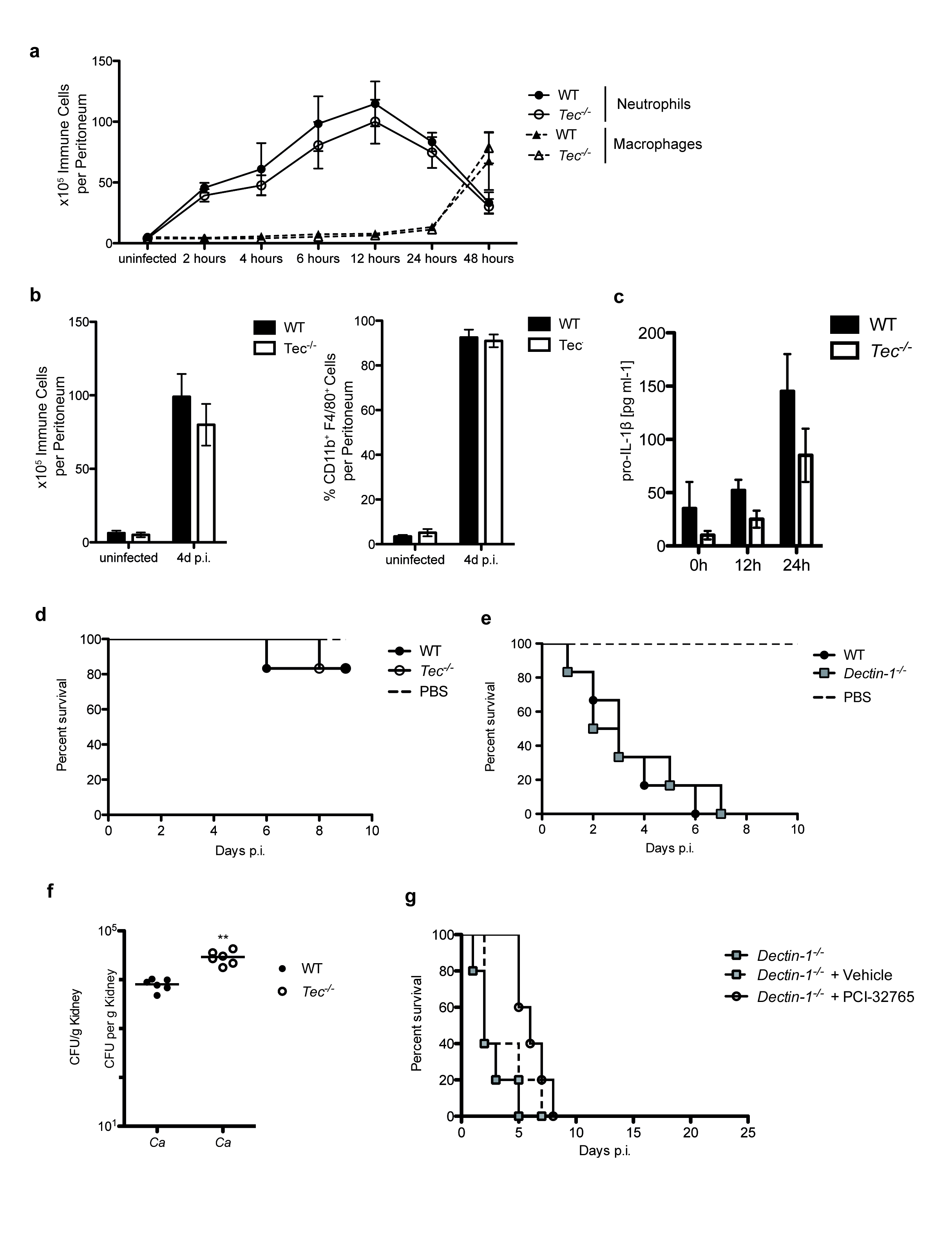

Supplement: Figure S8 — (a) Cell numbers of recruited peritoneal cells upon intraperitoneal infection (i.p.) with 5.106 CfUs of C. albicans after indicated time of infection or cells from uninfected mice; Cell type was assessed by FACS; Neutrophils: CD11b+Ly6G+F4/80−, Macrophage: CD11b+F4/80+; n = 3 per genotype and time point. (b) Cell numbers of recruited peritoneal cells upon intraperitoneal infection (i.p.) with 3% brewer thioglykolate medium for 4 days or cells from uninfected mice; Cell type was assessed by FACS; peritoneal macrophages: CD11b+F4/80+; n = 4 per genotype and time point. (c) ELISA of pro-IL-1β in cell lysates of peritoneal cells after indicated time of infections. (d) Survival of mice after intraperitoneal infection (i.p.) with 1.107 CfUs of C. albicans. n = 6 per genotype (e) Survival of mice after intraperitoneal infection (i.p.) with 5.107 CfUs of C. albicans.; for analysis of mouse survival curves Log-rank (Mantle-Cox) test was used. n = 6 per genotype. (f) Fungal loads in kidneys of mice after intravenous infection (i.v.) with 1.105 CfUs of C. albicans 7 days post infection; n = 6 per genotype. Data are representative of at least three (a–c) independent experiments. Mean and SD are shown (a–c). (TIFF) [file ppat.1004525.s008.tiff]

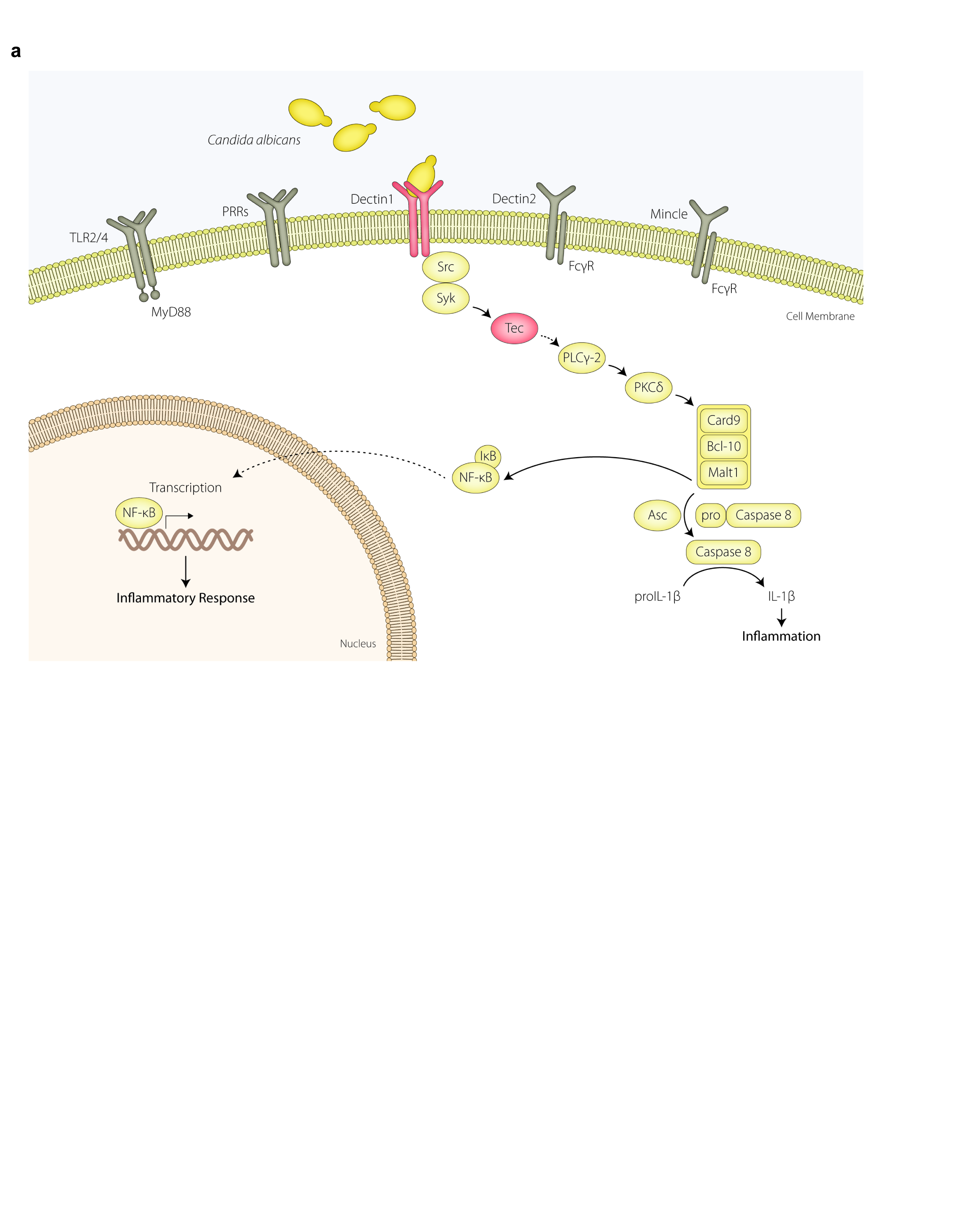

Supplement: Figure S9 — (a) Model. (TIFF) [file ppat.1004525.s009.tiff]

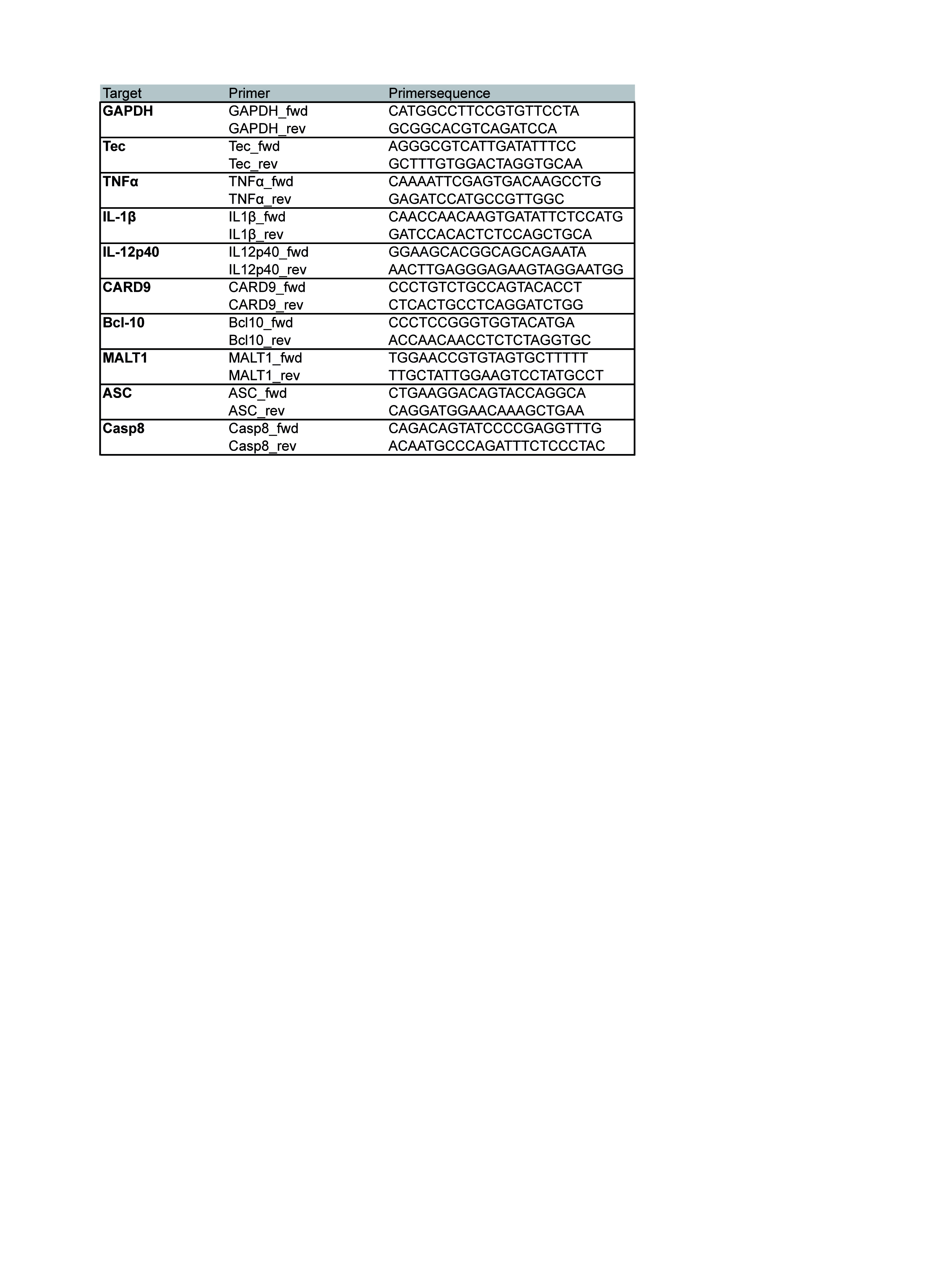

Supplement: Table S1 — Sequences of real-time primers used in this study. (TIFF) [file ppat.1004525.s010.tiff]
